# Supplementary material for: DCAlign v1.0: aligning biological sequences using co-evolution models and informed priors
Source: Bioinformatics. 2023 Aug 30;39(9):btad537. doi: 10.1093/bioinformatics/btad537 (PMC10491954; doi:10.1093/bioinformatics/btad537)
Supplement: btad537_Supplementary_Data [file btad537_supplementary_data.pdf]

Supplementary data for
DCAlign v1.0: Aligning biological sequences using co-evolution models and informed priors

Anna Paola Muntoni[1,2] and Andrea Pagnani[1,2,3]

[1]*Politecnico di Torino, Corso Duca degli Abruzzi, 24, I-10129, Torino, Italy*
[2]*Italian Institute for Genomic Medicine, IRCCS Candiolo, SP-142, I-10060 Candiolo (TO), Italy*
[3]*INFN, Sezione di Torino, Torino, Italy*

CONTENTS

I. Supplementary text 1
A. Modeling alignments 1
B. Standard learning of the gap penalties 2
C. Empirical prior over the pointer-difference variables 3
D. Approximate message-passing equations 4
E. Annealing scheme 5
F. Computation of the Positive Predicted Value curves 5
G. Computation of the proximity histograms 6

II. Supplementary results 7
A. Description of the protein and RNA domains 7
B. Positive predictive value curves 9
C. Proximity histograms 13
D. Leave-one-out simulations 17
1. BAliBASE 17
2. BRaliBase III 22
3. PH domain 24
4. Other structural-based alignments 26
E. Detection of distant sequences 28
F. Running time 30

References 31

I. SUPPLEMENTARY TEXT

A. Modeling alignments

In the following, we briefly summarize how to model aligned sequences according to `DCAlign`. Given the notation introduced in the main text, to obtain an ordered sequence suffices to impose *local* constraints on the variables $(\boldsymbol{x}, \boldsymbol{n})$ through a compatibility function $\chi_{i-1,i}\left(x_{i-1}, n_{i-1}, x_i, n_i\right)$ equals to one only for feasible assignments, namely

$$\begin{aligned}
\chi_{i-1,i}\left(0, n_{i-1}, 0, n_i\right) &= \mathbb{I}\left[n_i = n_{i-1}\right] \\
\chi_{i-1,i}\left(1, n_{i-1}, 0, n_i\right) &= \mathbb{I}\left[n_i = n_{i-1} \vee n_i = N+1\right] \\
\chi_{i-1,i}\left(0, n_{i-1}, 1, n_i\right) &= \mathbb{I}\left[0 \leq n_{i-1} < n_i < N+1\right] \\
\chi_{i-1,i}\left(1, n_{i-1}, 1, n_i\right) &= \mathbb{I}\left[0 < n_{i-1} < n_i < N+1\right]
\end{aligned} \tag{1}$$

where $\mathbb{I}\left[\mathcal{E}\right]$ is the indicator function of event $\mathcal{E}$. The original problem can be cast into a constrained optimization problem over the Boolean and pointer variables as

$$(\boldsymbol{x}, \boldsymbol{n}) = \arg\max_{(\bar{\boldsymbol{x}}, \bar{\boldsymbol{n}})} Z\left(\beta\right)^{-1} e^{-\beta \tilde{\mathcal{H}}(\bar{\boldsymbol{x}}, \bar{\boldsymbol{n}})} \prod_{i,i+1} \chi_{i,i+1}\left(\bar{x}_i, \bar{n}_i, \bar{x}_{i+1}, \bar{n}_{i+1}\right) \tag{2}$$

where Z is the partition function, i.e. the normalization of the joint distribution of the variables

$$Z(\beta) = \sum_{\substack{\boldsymbol{x},\boldsymbol{n}: \\ \prod_{i,i+1}\chi_{i,i+1}=1}} e^{-\beta\tilde{\mathcal{H}}(\boldsymbol{x},\boldsymbol{n})} \tag{3}$$

and $\tilde{\mathcal{H}}$ is (minus) the objective function, namely the Hamiltonian characterizing the seed

$$\tilde{\mathcal{H}}(\boldsymbol{x},\boldsymbol{n}) = \mathcal{H}_{\mathrm{DCA}}(\boldsymbol{x},\boldsymbol{n}) - \mathcal{H}_{\mathrm{ins}}(\boldsymbol{x},\boldsymbol{n}) - \mathcal{H}_{\mathrm{gap}}(\boldsymbol{x},\boldsymbol{n}) \tag{4}$$

The term $\mathcal{H}_{\mathrm{DCA}}(\boldsymbol{x},\boldsymbol{n})$ is the Potts Hamiltonian learned from the seed sequences using standard inverse modeling procedures

$$\mathcal{H}_{\mathrm{DCA}}(\boldsymbol{x},\boldsymbol{n}) = -\sum_{i,j} J_{i,j}\left(A_{x_i n_i}, A_{x_j n_j}\right) - \sum_i h_i\left(A_{x_i n_i}\right) \tag{5}$$

where $A_0 =$ '$-$'. More precisely, to infer the coupling matrices and the fields, we employ Boltzmann machine learning [11] for the algorithms denoted as `DCAlign`, `DCAlign v1.0 (bm)` while for the new implementation encoded into `DCAlign v1.0 (plm)` we use pseudo-likelihood maximization [4].

The other two energy terms $\mathcal{H}_{\mathrm{ins}}$ and $\mathcal{H}_{\mathrm{gap}}$ penalize the presence of insertions or gaps within the aligned sequences. They take the form of

$$\mathcal{H}_{\mathrm{ins}}(\boldsymbol{x},\boldsymbol{n}) = \sum_i \left(1-\delta_{\Delta n_i,0}\right)\left[\lambda_o^i + \lambda_e^i\left(\Delta n_i - 1\right)\right] \tag{6}$$

$$\mathcal{H}_{\mathrm{gap}}(\boldsymbol{x},\boldsymbol{n}) = \sum_i \delta_{x_i,0}\{\mu^{\mathrm{int}}\mathbb{I}\left[0 < n_i < N+1\right] + \mu^{\mathrm{ext}}\mathbb{I}\left[n_i = 0 \vee n_i = N+1\right]\} \tag{7}$$

where Δn_i is the number of insertions between positions i and $i-1$, λ_o^i (λ_e^i) is the penalty for creating (adding) an insertion at position i and μ^{ext} (μ^{int}) is the cost of using a gap symbol at the beginning or the end of the aligned sequence (within two symbols). While the parameters $\{\boldsymbol{\lambda}_o, \boldsymbol{\lambda}_e\}$ can be learned by means of a (fast) likelihood maximization, the learning of the gap penalties μ^{ext} and μ^{int} are determined in a supervised and slow manner (see Sec. I B). To significantly decrease the computation time necessary to determine $\mathcal{H}_{\mathrm{ins}}$ and $\mathcal{H}_{\mathrm{gap}}$, we introduce in Sec. I C an empirical prior which accommodates both the presence of insertions and gaps.

B. Standard learning of the gap penalties

As discussed in [10], if the seed alignment contains a large number of sequences ($\geq 10^3$), it suffices to re-align all of them for each set of candidate gap penalties $\left(\mu^{\mathrm{int}}, \mu^{\mathrm{ext}}\right)$ and to choose those that minimize the average Hamming distance between the original seed sequences and the re-aligned ones. The trial number of gap penalties is often about 80, for $\mu^{\mathrm{int,\,ext}} \in [0.0, 4.0]$ which coincides with the number of times the seed needs to be re-aligned. Considering that the computation time is on average of a few seconds for a sequence of length N and the number of sequences is about one hundred, the overall time is of one day.

Instead, if the seed is composed of a few sequences (i.e. less than 10^3), the average Hamming distance is always zero for all candidate gap penalties, i.e. the re-aligned sequences always coincide with the true sequences, carrying no information about the best set of parameters. To resort to this issue, it is reasonable to introduce a validation set of sequences to be aligned using all candidate gap penalties (usually of the order of 10^3). Alternatively to the Hamming distance, one may consider the symmetric Kullback-Leibler divergence sDKL between a seed model $\mathcal{H}_{\mathrm{DCA}}$ and a model learned from each of the candidate aligned test set obtained for a couple $\left(\mu^{\mathrm{ext}}, \mu^{\mathrm{int}}\right)$, that is $\mathcal{H}_{\mathrm{DCA}}^{\left(\mu^{\mathrm{ext}},\,\mu^{\mathrm{int}}\right)}$, defined as

$$\mathrm{sDKL} = \left\langle \mathcal{H}_{\mathrm{DCA}} - \mathcal{H}_{\mathrm{DCA}}^{\left(\mu^{\mathrm{ext}},\mu^{\mathrm{int}}\right)} \right\rangle_{\mathcal{H}_{\mathrm{DCA}}^{\left(\mu^{\mathrm{ext}},\mu^{\mathrm{int}}\right)}} + \left\langle \mathcal{H}_{\mathrm{DCA}}^{\left(\mu^{\mathrm{ext}},\mu^{\mathrm{int}}\right)} - \mathcal{H}_{\mathrm{DCA}} \right\rangle_{\mathcal{H}_{\mathrm{DCA}}} \tag{8}$$

Here the symbol $\langle a \rangle_b$ denotes the average value of the quantity a according to the Boltzmann distribution having Hamiltonian b. This last step not only requires aligning a huge number of sequences (as for the copious seed case), but the computation of the Kullback-Leibler distance requires learning as many models as the number of candidate gap penalties, and sampling from them (to compute the expectation values) using Monte Carlo Markov Chain.

Considering that, using Boltzmann machine learning, the learning of a DCA model costs several hours of computation, the overall process necessitates a computational time that varies from one day to several days.

Unaligned sequence FGYLAKADTL

1 2 3 4 5 6 7 8 9 10 n

Hybrid format f**GY**lak**AD--**t**L**

Aligned sequence **GYAD--L**

1 2 3 4 5 6 7 i

Assignment associated with the aligned seq. $\boldsymbol{n}^{\star} = (2,3,7,8,8,8,10)$

$\boldsymbol{x}^{\star} = (1,1,1,1,0,0,1)$

$i \backslash j$	2	3	4	5	6	7
1	1 Y	5 YLAKA	6 YLAKAD	6 YLAKAD	6 YLAKAD	8 YLAKADTL
2		4 LAKA	5 LAKAD	5 LAKAD	5 LAKAD	7 LAKADTL
3			1 D	1 D	1 D	3 DTL
4				0	0	2 TL
5					0	2 TL
6						2 TL

FIG. S1. In the left panel an unaligned sequence of length $N = 10$ is proposed as well as the associated aligned sequence of length $L = 7$; the indices n and i run over all the possible values, as indicated below each symbol. For the sake of clarity an alignment in a hybrid format (where matched, insertions and gaps are present), is shown between the two. The two vectors $\boldsymbol{n}^{\star}$ and $\boldsymbol{x}^{\star}$ take value according to the aligned sequences. From $\boldsymbol{n}^{\star}$, we compute in the table (right panel), all possible values for the $\Delta n_{i,j}$ where $i = 1, \ldots, 6$ (in the rows), and $j = 2, \ldots, 7$ (in the columns). Each entry contains $\Delta n_{i,j} = n^{\star}_j - n^{\star}_i$ followed by the symbols, of the unaligned sequence, between the positions i and j (rows and columns of the table respectively) of the aligned one, whose counting is equal to $\Delta n_{i,j}$; by convention, we consider in the counting the last symbol $n^{\star}_j$ and we neglect $n^{\star}_i$. This last rule allows us to discriminate when gaps (even stretches of gaps) are present, i.e. $\Delta n_{i,j} = 0$, and when this is not the case, $\Delta n_{i,j} > 0$. When $\Delta n_{i,j} > 0$ both matched symbols and insertions contribute.

C. Empirical prior over the pointer-difference variables

A much faster strategy aimed at modeling the presence of gaps and insertions exploits the information over the realization of the pointer variables $\boldsymbol{n}$ in the seed sequences.
As described in the main text, for each couple of indices of the columns of the MSA, let us denote them as i and $j > i$, we compute the empirical frequencies (over the seed sequences) of the corresponding $\Delta n_{i,j} = n_j - n_i$. This variable quantifies the number of symbols that are present, within the unaligned sequence, between the positions i and j of the aligned counterpart. For the sake of clarity, let us consider the $j = i+1$ case; here $j - i = 1$, and depending on the value $\Delta n_{i,i+1}$ we can identify three scenarios. $\Delta n_{i,i+1} = 1 = j - i$ reveals that no insertion and no gap are present between i and $i+1$ because the symbols appearing in the aligned sequence are consecutive in the unaligned one. Instead, if $\Delta n_{i,i+1} > 1$ more than one symbol lies between A_{n_i} and $A_{n_{i+1}}$ and, in this case, $\Delta n_{i,i+1} - 1$ quantifies the number of insertions between i and $i+1$. Conversely, if $\Delta n_{i,i+1} = 0 < j - i$ the two pointers n_i and n_{i+1} coincide and this is only possible when a gap appears in $A_{n_{i+1}}$. For a generic case, $j > i+1$, the three situations may overlap meaning that both insertions and matched symbols contribute to the computation of the pointer differences. When, instead, $\Delta n_{i,j} = 0$ between the columns i and j only gaps appear. An example is shown in Fig.S1.

Independently of the nature of these contributions, adding an empirical prior over all possible $i < j$ to the objective function informs the pointer variables to be inferred about their statistical behavior within the seed sequences.
Let us define $\tilde{\Lambda}_{i,j}(\Delta n)$ the normalized histogram of the pointer-difference variables associated with columns i and j. Note that the number of possible argument Δn of the empirical probability $\tilde{\Lambda}_{i,j}(\Delta n)$ depends on the length of the longest sequence of the seed. Before adding the empirical probability to the objective function in Eq. 2, we need to ensure that, once we align a sequence of length N, the maximum possible value $\Delta n = N + 1 - 0$ is covered by the empirical prior. Similarly, being $\tilde{\Lambda}(\Delta n)$ obtained from (usually) a few sequences, some arguments may have exactly zero probability. This would prevent several assignments that may be, although improbable, the best choice for the target pointer variables. For this reason, we re-weight the empirical statistics adding a small pseudo-count equals to $1/M_{seed}$ for the unobserved states (where M_{seed} is the number of seed sequences). The probability thus obtained is

$$\Lambda_{i,j}(\Delta n) \propto \left(1 - \frac{1}{M_{seed}}\right) \tilde{\Lambda}_{i,j}(\Delta n) + \frac{1}{M_{seed}}, \quad \Delta n \in [0, \ldots, N+1] \tag{9}$$

where the proportionality symbol accounts for the missing normalization ensuring that $\sum_{\Delta n} \Lambda_{i,j}(\Delta n) = 1$. The alignment problem is therefore re-phrased in `DCAlign v1.0` as

$$(\boldsymbol{x}, \boldsymbol{n}) = \arg\max_{(\bar{\boldsymbol{x}}, \bar{\boldsymbol{n}})} Z(\beta)^{-1} e^{-\beta \mathcal{H}_{\mathrm{DCA}}(\bar{\boldsymbol{x}}, \bar{\boldsymbol{n}})} \prod_{\substack{i,j: \\ j>i}} \Lambda^{\beta}_{i,j}(\Delta \bar{\boldsymbol{n}}) \prod_{i,i+1} \chi(\bar{x}_i, \bar{n}_i, \bar{x}_{i+1}, \bar{n}_{i+1}) \tag{10}$$

$i=1$	$i=2,\cdots,L-1$	$i=L$
$m_1=\frac{1}{z_1}\mathcal{C}_1\mathcal{B}_1$	$m_i=\frac{1}{z_i}\mathcal{C}_i\mathcal{F}_i\mathcal{B}_i$	$m_L=\frac{1}{z_L}\mathcal{C}_L\mathcal{F}_L$
$F_1=\frac{1}{f_1}\mathcal{C}_1$	$F_i=\frac{1}{f_i}\mathcal{C}_i\mathcal{F}_i$	—
—	$B_i=\frac{1}{b_i}\mathcal{C}_i\mathcal{B}_i$	$B_L=\frac{1}{b_L}\mathcal{C}_L$

TABLE S1. Sketch of the message-passing update equations. The terms $\mathcal{F}_i$, $\mathcal{B}_i$, $\mathcal{C}_i$ are given in Eqs. 13, 14 and 15.

D. Approximate message-passing equations

A brute-force solution to the optimization problem in Eq. 10 would require computing the partition function

$$Z(\beta)=\sum_{\substack{\boldsymbol{x},\boldsymbol{n}:\\ \prod_{i,i+1}\chi_{i,i+1}=1}} e^{-\beta\mathcal{H}_{\mathrm{DCA}}(\boldsymbol{x},\boldsymbol{n})}\prod_{\substack{i,j:\\ j>i}}\Lambda_{i,j}^{\beta}(\Delta\boldsymbol{n}) \tag{11}$$

where the hard constraint $\prod_{i,i+1}\chi_{i,i+1}=1$ is indicated under the first sum. This is a computationally hard problem as the number of terms involved in the sum scales as $\mathcal{O}\left((N+2)^L\right)$. In `DCAlign`, an approximation of the marginal probability densities $m_i(x_i,n_i)$ of the joint distribution of the variables (whose normalization appears in Eq. 3) are retrieved by iterating a set of simplified message-passing equations. From them, an assignment of the variables, and therefore the aligned sequence, is determined by looking at the arguments that maximize the marginal probabilities

$$(x_i,n_i)=\arg\max_{x,n} m_i(x,n) \qquad i=1,...,L. \tag{12}$$

In cases where the assignment does not satisfy the hard constraints encoded in the χ-functions, it is possible to resort to a *nucleation* algorithm (see [10]).

Similarly to the first implementation, in `v1.0`, the general update scheme can be expressed using a transfer-matrix formulation (see Table S1). The introduction of the empirical prior slightly modifies the *forward*, *backward* and *central* terms as

$$\begin{aligned}
\mathcal{F}_i(x_i,n_i) &= \sum_{x_{i-1},n_{i-1}} F_{i-1}(x_{i-1},n_{i-1})\, e^{\beta J_{i-1,i}\left(A_{x_{i-1}n_{i-1}},A_{x_i n_i}\right)}\times \\
&\quad \times\chi_{i-1,i}(x_{i-1},n_{i-1},x_i,n_i)\,\Lambda_{i-1,i}^{\beta}(n_i-n_{i-1})
\end{aligned} \tag{13}$$

$$\begin{aligned}
\mathcal{B}_i(x_i,n_i) &= \sum_{x_{i+1},n_{i+1}} B_{i+1}(x_{i+1},n_{i+1})\, e^{\beta J_{i+1,i}\left(A_{x_i n_i},A_{x_{i+1}n_{i+1}}\right)}\times \\
&\quad \times\chi_{i,i+1}(x_i,n_i,x_{i+1},n_{i+1})\,\Lambda_{i,i+1}^{\beta}(n_{i+1}-n_i)
\end{aligned} \tag{14}$$

$$\begin{aligned}
\mathcal{C}_i(x_i,n_i) &= \exp\Big[\beta h_i\left(A_{x_i n_i}\right)+ \\
&\quad +\sum_{j<i-1}\sum_{x_j,n_j}\chi_{lr}(x_i,n_i,x_j,n_j)\,\beta J_{i,j}\left(A_{x_i n_i},A_{x_j n_j}\right) m_j(x_j,n_j)\,\Lambda_{i,j}^{\beta}(\Delta n_{i,j})+ \\
&\quad +\sum_{j>i+1}\sum_{x_j,n_j}\chi_{lr}(x_i,n_i,x_j,n_j)\,\beta J_{i,j}\left(A_{x_i n_i},A_{x_j n_j}\right) m_j(x_j,n_j)\,\Lambda_{j,i}^{\beta}(\Delta n_{j,i})\Big]
\end{aligned} \tag{15}$$

The χ_{lr} are redundant 'long-range' compatibility functions involving non-neighbor sites

$$\begin{aligned}
\chi_{lr}(x_i,n_i,x_j,n_j) &= \mathbb{I}[i>j+1]\left\{\delta_{x_i,0}\mathbb{I}[n_i\geq n_j]+\delta_{x_i,1}\mathbb{I}[n_i>n_j]\right\}+ \\
&\quad \mathbb{I}[i<j-1]\left\{\delta_{x_j,0}\mathbb{I}[n_i\leq n_j]+\delta_{x_j,1}\mathbb{I}[n_i<n_j]\right\}
\end{aligned} \tag{16}$$

derived from the short-range ones. Eqs. 13, 14 and 15 may be iteratively updated, together with those in Table S1, by setting the inverse temperature $\beta=1$, up to a numerical convergence. This temperature could be understood as the 'natural' temperature of the system fully determined by the inverse methods used to parametrize the seed sequences. In `DCAlign` this scheme was sufficient for obtaining concentrated marginals and therefore a straightforward decoding to get the aligned sequence. However, in `v1.0`, at numerical convergence, very often the marginal probabilities $m_i(x_i,n_i)$ are dispersed over several states signaling a coexistence of energetically equivalent alignments, at $\beta=1$. This renders the final assignment of the variables hard, as the naif maximization of the marginal probabilities may lead to an unfeasible assignment (see Fig. S2). To solve this issue, and to actually implement the maximization step in Eq. 10, we resort to an annealing scheme over β.

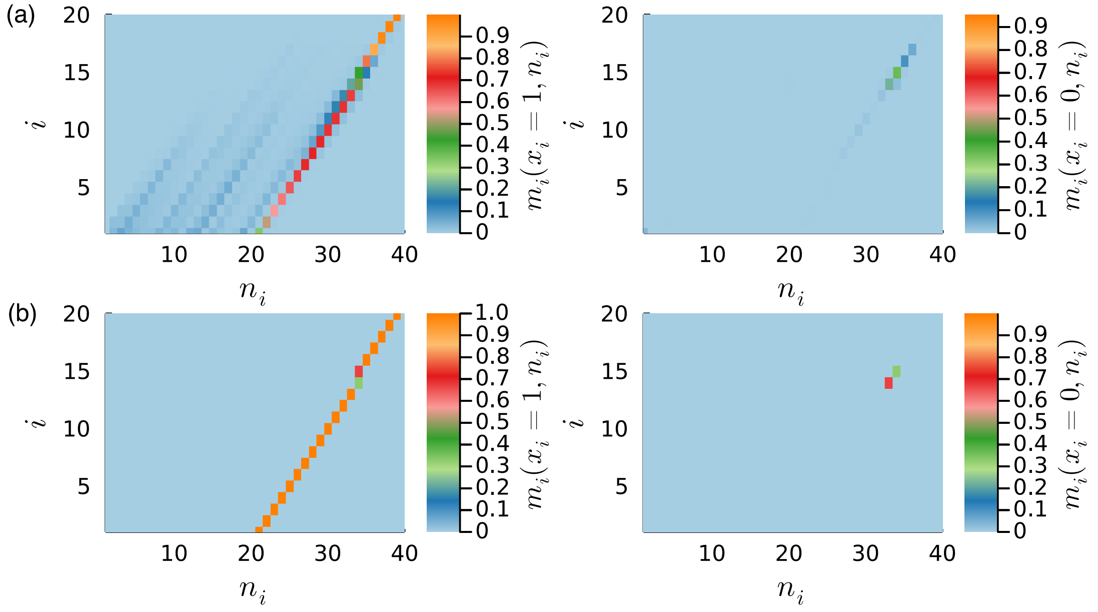

FIG. S2. Panel (a) shows the marginal probabilities $m_i(x_i = 1, n_i)$ (right panel) and $m_i(x_i = 0, n_i)$ (left panel) for the sites $i = 1, \ldots, 20$, and $n_i = 0, \ldots, 40$ when the message-passing equations are iterated at $\beta = 1$. Panel (b) shows instead how they change when we perform the annealing over β. Probabilities for $i < 20$ appear concentrated in panel (b) rendering the assignment step straightforward. The target protein domain belongs to PF00035 and appears within the protein `A0A2U9BW26_SCOMX`.

E. Annealing scheme

To allow the message-passing dynamics to polarize on (one of) the best possible assignment of the variables, we increase the inverse temperature β within the iterations of the equations, i.e. each $\Delta t = 10$ iterations β is increased of $\Delta\beta = 0.05$. We check the polarization of the marginals by looking at the minimum value, among the sites, of the maximum value, over the states, of the $m_i(x_i, n_i)$. When this number is larger than a threshold and the assignment retrieved from the arguments that maximize the marginal probabilities is feasible, we stop the algorithm. An example is reported in Fig. S2 for a sequence belonging to Pfam PF00035. We show in panel (a) the results for $\beta = 1$ while in panel (b) we display the marginals obtained through the annealing scheme. The left (right) plot shows the conditional probability for a subset of sites $i \in [1, 20]$ to be aligned to position $n_i \in [0, 40]$ for $x_i = 1$ (for $x_i = 0$). In Fig. S2 panel (a) the marginal probability is dispersed over several values of n_i for $i < 20$. In particular, the assignment provided by the maximization of the marginals is unfeasible because the sites $i = 14, 15$ both point to $n = 33$. The issue is solved using the annealing over β as displayed in panel (b): site $i = 14$ is now pointing to $n_{14} = 33$ for $x_{14} = 1$ while in $i = 15$ a gap appears. In this example, we choose a threshold of 0.7, however, we empirically observe that 0.30 suffices to guarantee polarized and feasible assignment.

F. Computation of the Positive Predicted Value curves

To verify whether the introduction of the empirical prior affects the quality of the alignment, we compare the outcomes provided by `v1.0` to those obtained by `DCAlign` and state-of-the-art techniques. The idea is to infer a DCA model (learned using pseudo-likelihood maximization) for each candidate multiple sequence alignment whose parameters are used to perform a contact prediction of the associated domain. In particular, for each DCA model, we compute the set of average product corrected Frobenius norms of the coupling matrices [4] which quantify how likely the pairs of sites are in contact according to the model.

To evaluate the quality of the prediction, we use as ground truths the distance map provided by Pfam interactions package [14] which considers several experimentally obtained structures downloaded from the Protein Data Bank [3]. Note that to allow for any comparison, we first need to map the residue sites of the crystallized sequence to those of the multiple sequence alignment. In principle, this mapping may change depending on the used alignment tool. For the sake of simplicity, we use the mapping provided by Pfam interactions tool [14] and PdbTool [6] for protein and RNA domains respectively, which use the site-residue relationship provided by Pfam or Rfam. Then, using the sorted

Frobenius norms as scores, we compute the Positive Predictive Value (PPV) curve for each method. More details are shown in the caption of Figs. S3 and S4. A summary of the contact prediction results is also reported in Tabs. S7 and S8.

G. Computation of the proximity histograms

Together with the contact prediction we use a sequence-based metric to evaluate the quality of the new alignment scheme. The measure we consider consists of the distribution of the distances between the seed and the multiple sequence alignments obtained for the considered methods. Let us define as $\mathbf{S}^{seed}$ the set of the seed sequences and $\mathbf{S}^{MSA}$ the query sequences aligned by the considered methods. For each sequence S_j^{MSA} we compute the Hamming distance d_H between S_j^{MSA} and all the seed sequences and we keep the minimum distance

$$d_j = \min_i d_H \left(S_j^{MSA}, S_i^{seed} \right) \tag{17}$$

Then, we plot the histogram of the minimal distances $\mathbf{d}$ achieved by each method. The more the mass of the empirical distribution is concentrated at low distances, the more the aligned sequences are similar to the seed.

II. SUPPLEMENTARY RESULTS

A. Description of the protein and RNA domains

Identifier	M_{train}	M_{test}	L	PDBs	Ref.
PF00035	81	10000	67	73	Pfam v32.0
PF00677	1878	14683	87	9	Pfam v32.0
PF00684	1512	10000	67	3	Pfam v32.0
PF00763	1389	10000	116	24	Pfam v32.0
PF00062	$M_{\text{test}} - 1$	PDBs	117	1127	PDB and Caretta
PF00182	$M_{\text{test}} - 1$	PDBs	198	27	PDB and Caretta
PF00638	$M_{\text{test}} - 1$	PDBs	185	58	PDB and Caretta
PF00640	$M_{\text{test}} - 1$	PDBs	56	40	PDB and Caretta
PF01464	$M_{\text{test}} - 1$	PDBs	77	61	PDB and Caretta
PF09380	$M_{\text{test}} - 1$	PDBs	253	40	PDB and Caretta
PF00013	$M_{\text{test}} - 1$	PDBs	62	92	PDB and Caretta
PF09408	$M_{\text{test}} - 1$	PDBs	81	1429	PDB and Caretta
IPR000008	$M_{\text{test}} - 1$	PDBs	158	204	PDB and Caretta
IPR000020	$M_{\text{test}} - 1$	PDBs	60	44	PDB and Caretta
IPR001466	$M_{\text{test}} - 1$	PDBs	226	321	PDB and Caretta
IPR001789	$M_{\text{test}} - 1$	PDBs	64	436	PDB and Caretta

TABLE S2. Pfam [5, 9] and InterPro [12] domains used in this work (if not differently stated, we assume to use Pfam v33 [9]). Along with the identifier, we show the number of sequences used to learn a DCA model, M_{train}, those to be aligned M_{test}, the length L of the domain, the number of crystallized structures available in the Protein Data Bank PDBs and the online repositories used to download or pre-process the data.
In particular, the first four protein families are those studied in [10] and used here as a comparison in the contact map prediction and proximity measure histograms. The number M_{train} coincides here with the number of sequences in the seed alignments.
The next six families belong to the Pfam clan CL0266 containing PH-type domains, while the last six have been chosen for having a reasonable number of known crystallized structures. These identifiers are used to collect and structurally align the sequences of the known structures available in the Protein Data Bank. The alignments, performed by Caretta [1], are used as ground truth in the leave-one-out test, prior to the identification of the reference columns to prevent gappy multiple sequence alignments. The number of sequences used as a test equals the number of available PDBs and the number of sequences used in the training is always set to $M_{\text{test}} - 1$.

Identifier	M_{train}	M_{test}	L	PDBs	Ref.
RF00059	109	12593	105	24	Rfam v13.0
RF00162	433	6026	108	25	Rfam v13.0
RF00167	133	2660	102	49	Rfam v13.0
RF01734	287	2138	63	6	Rfam v13.0

TABLE S3. Rfam domains used in this work. Along with the identifier, we show the number of sequences used to learn a DCA model for the seed, M_{train}, those to be aligned M_{test}, the length L of the domain, the number of crystallized structures available in the Protein Data Bank PDBs and the online repository used to download the data. The four RN families are those studied in [10] and used here as a comparison in the contact map prediction and proximity measure histograms. The number M_{train} coincides here with the number of sequences in the seed alignments.

Identifier	M_{train}	M_{test}	L	Ref.	Identifier	M_{train}	M_{test}	L	Ref.
BBS20008	$M_{\text{test}} - 1$	56	79	BAliBASE 4	BBS20038	$M_{\text{test}} - 1$	42	91	BAliBASE 4
BBS20030	$M_{\text{test}} - 1$	47	88	BAliBASE 4	BBS30001	$M_{\text{test}} - 1$	116	167	BAliBASE 4
BBS20032	$M_{\text{test}} - 1$	60	95	BAliBASE 4	BBS30020	$M_{\text{test}} - 1$	56	58	BAliBASE 4
BBS20033	$M_{\text{test}} - 1$	48	96	BAliBASE 4	BBS30022	$M_{\text{test}} - 1$	66	77	BAliBASE 4
BBS20036	$M_{\text{test}} - 1$	91	71	BAliBASE 4	BBS30025	$M_{\text{test}} - 1$	74	72	BAliBASE 4

TABLE S4. Reference sets selected from BAliBASE 4 [2] [16] for the leave-one-out test. These collections optimally trade-off between the number of sequences M and the length L of the domain (as $L \sim M$), considering that the number of parameters of the Potts model, i.e. $O\left(L^2\right)$, have to be learned from M sequences. Sequence sets having $M \ll L$ have been not considered for this reason.

Identifier	M_{train}	M_{test}	L	Ref.
rRNA	$M_{\text{test}} - 1$	602	119	BRaliBase III
tRNA	$M_{\text{test}} - 1$	1114	72	BRaliBase III
U5	$M_{\text{test}} - 1$	235	118	BRaliBase III

TABLE S5. Reference sets of RNA alignments taken from BRaliBase III [7] and used in the leave-one-out test.

Identifier	M_{train}	M_{test}	L	Ref.
type P1	1474	139	65	[13]
tRNA	963	452	71	[15]
RF00162 seed	319	137	108	[8]

TABLE S6. Reference sets of the RNA alignments considered in [17] and clusterized in the train-test experiment. See Fig. S13 and Tab. S15.

B. Positive predictive value curves

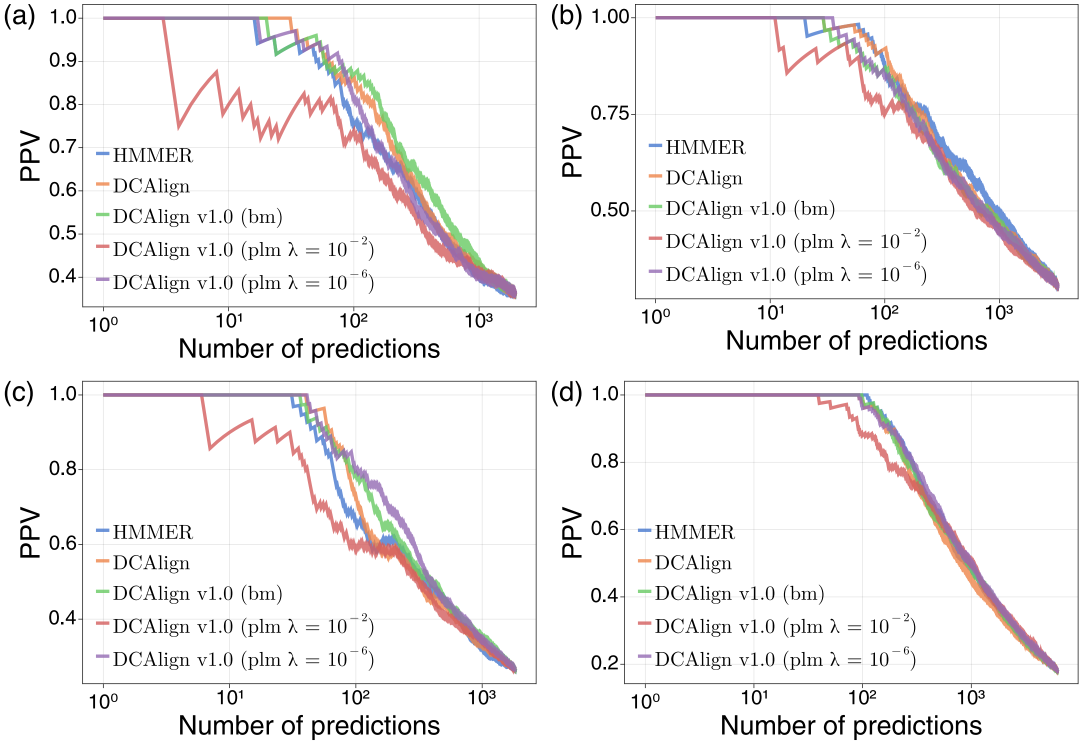

FIG. S3. Positive predictive value (PPV) curves associated with the prediction of the three-dimensional structure of the Pfam domains PF00035, PF00677, PF00684, and PF00763 in panels (a), (b), (c), and (d) respectively.
From each multiple sequence alignment provided by `HMMER`, `DCAlign`, `DCAlign v1.0`, we learn a Potts model using pseudo-likelihood maximization and we compute the average product corrected Frobenius norms of the coupling matrices. The latter is used as a proxy for the probability of the sites of being in contact within the three-dimensional structure of the domains. For this comparison, we consider only the predictions over non-trivial pairs of sites, i.e. couples i and j sufficiently far in the chain, such that $|i - j| > 4$. The distance maps used as ground truth, as well as the mapping between the MSA columns and residue sites, are provided by running Pfam interactions [14] and consist of the minimum distances, computed among several crystallized structures, between any atom of the considered residue pairs. A contact is established if such a distance is smaller than 8 Å.
For DCAlign v1.0 we report the predictions achieved by three multiple sequence alignments differing in the seed model and used to align the test sequences. The label (bm) or (plm) refers to the method used to determine the parameters of the Potts model; for (plm) only we consider two values for the parameter λ governing the regulation strength of both fields and couplings.

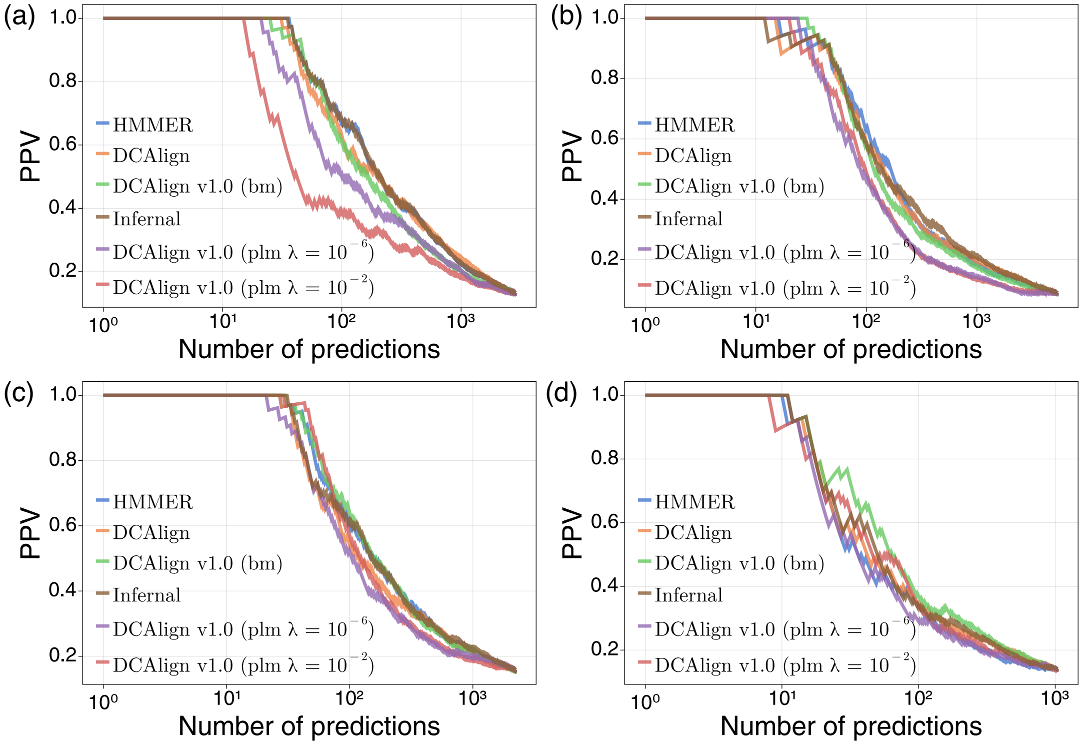

FIG. S4. Positive predictive value (PPV) curves associated with the prediction of the three-dimensional structure of the Rfam domains RF00059, RF00162, RF00167, and RF01734 in panels (a), (b), (c), and (d) respectively.
From each multiple sequence alignment provided by `HMMER`, `DCAlign`, `DCAlign v1.0`, and `Infernal`, we learn a Potts model using pseudo-likelihood maximization and we compute the average product corrected Frobenius norms of the coupling matrices. The latter is used as a proxy for the probability of each pair of sites of being in contact within the three-dimensional structure of the domain. For this comparison, we consider only the predictions over non-trivial pairs of sites, i.e. couples i and j sufficiently far in the chain, such that $|i-j| > 4$. The distance map is determined as follows. For each available structure, we first run PdbTool [6] to (i) compute the residue pairwise distances as the distance between the two closest heavy atoms in the residues and (ii) to map each MSA position to a residue of the structure by aligning the sequence to the covariance model (CM) associated with the seed and available in Rfam. The final distance map is obtained by the minimal distances computed over the set of known structures. As for the PPV obtained for the protein families, we consider two sites in contact if their distance is smaller than 8 Å.
For DCAlign v1.0 we report the predictions achieved by three multiple sequence alignments differing in the seed model and used to align the test sequences. The label (bm) or (plm) refers to the method used to determine the parameters of the Potts model; for (plm) only we consider two values for the parameter λ governing the regulation strength of both fields and couplings.

	PF00035		
	First FP	TPR(2L)	TPR $<$ 0.80
DCAlign v1.0 (plm $\lambda = 10^{-2}$)	4	0.664	4
DCAlign v1.0 (plm $\lambda = 10^{-6}$)	18	0.739	111
DCAlign v1.0 (bm)	21	**0.851**	**163**
DCAlign	**32**	0.806	144
HMMER	17	0.731	84
	PF00677		
	First FP	TPR(2L)	TPR $<$ 0.80
DCAlign v1.0 (plm $\lambda = 10^{-2}$)	12	0.713	68
DCAlign v1.0 (plm $\lambda = 10^{-6}$)	**36**	0.753	139
DCAlign v1.0 (bm)	30	0.759	133
DCAlign	30	**0.770**	**154**
HMMER	21	0.764	**154**
	PF00684		
	First FP	TPR(2L)	TPR $<$ 0.80
DCAlign v1.0 (plm $\lambda = 10^{-2}$)	7	0.582	42
DCAlign v1.0 (plm $\lambda = 10^{-6}$)	**42**	**0.761**	**99**
DCAlign v1.0 (bm)	37	0.724	94
DCAlign	41	0.627	87
HMMER	32	0.590	67
	PF00763		
	First FP	TPR(2L)	TPR $<$ 0.80
DCAlign v1.0 (plm $\lambda = 10^{-2}$)	40	0.772	171
DCAlign v1.0 (plm $\lambda = 10^{-6}$)	93	**0.858**	**289**
DCAlign v1.0 (bm)	99	0.819	258
DCAlign	93	0.828	253
HMMER	**111**	**0.858**	281

TABLE S7. Summary of the contact prediction results. We report here, for each considered multiple sequence alignment, three metrics associated with the PPV curves displayed in Fig. S3. In the columns, we report the position of the ranking where the first false positive prediction arises (First FP), the true positive rate computed at $2 \cdot L$, TPR(2L), and the position in the ranking where the true positive rate (TPR) is smaller than 0.80 for the first time. Bold numbers refer to the best performance in each metric. Note that the best-performing methods are either `HMMER` or `DCAlign`, `DCAlign v1.0` when $\lambda = 10^{-6}$.

	RF00059		
	First FP	TPR(2L)	TPR < 0.80
DCAlign v1.0 (plm $\lambda = 10^{-2}$)	16	0.324	21
DCAlign v1.0 (plm $\lambda = 10^{-6}$)	22	0.386	43
DCAlign v1.0 (bm)	26	0.438	61
DCAlign	32	0.495	52
HMMER	**37**	**0.500**	59
Infernal	36	**0.500**	**62**
	RF00162		
	First FP	TPR(2L)	TPR < 0.80
DCAlign v1.0 (plm $\lambda = 10^{-2}$)	21	0.278	42
DCAlign v1.0 (plm $\lambda = 10^{-6}$)	25	0.282	39
DCAlign v1.0 (bm)	**30**	0.352	59
DCAlign	16	0.375	57
HMMER	17	0.403	**61**
Infernal	13	**0.417**	59
	RF00167		
	First FP	TPR(2L)	TPR < 0.80
DCAlign v1.0 (plm $\lambda = 10^{-2}$)	30	0.358	**62**
DCAlign v1.0 (plm $\lambda = 10^{-6}$)	22	0.358	46
DCAlign v1.0 (bm)	31	**0.451**	59
DCAlign	28	0.392	43
HMMER	28	0.441	53
Infernal	**32**	0.446	46
	RF01734		
	First FP	TPR(2L)	TPR < 0.80
DCAlign v1.0 (plm $\lambda = 10^{-2}$)	9	0.286	**18**
DCAlign v1.0 (plm $\lambda = 10^{-6}$)	**12**	0.278	17
DCAlign v1.0 (bm)	**12**	**0.325**	**18**
DCAlign	**12**	0.310	**18**
HMMER	11	0.302	**18**
Infernal	**12**	0.302	**18**

TABLE S8. Summary of the contact prediction results. We report here, for each considered multiple sequence alignment, three metrics associated with the PPV curves displayed in Fig. S4. In the columns, we report the position of the ranking where the first false positive prediction arises (First FP), the true positive rate computed at $2 \cdot L$, TPR(2L), and the position in the ranking where the true positive rate (TPR) is smaller than 0.80 for the first time. Bold numbers refer to the best performance in each metric.

C. Proximity histograms

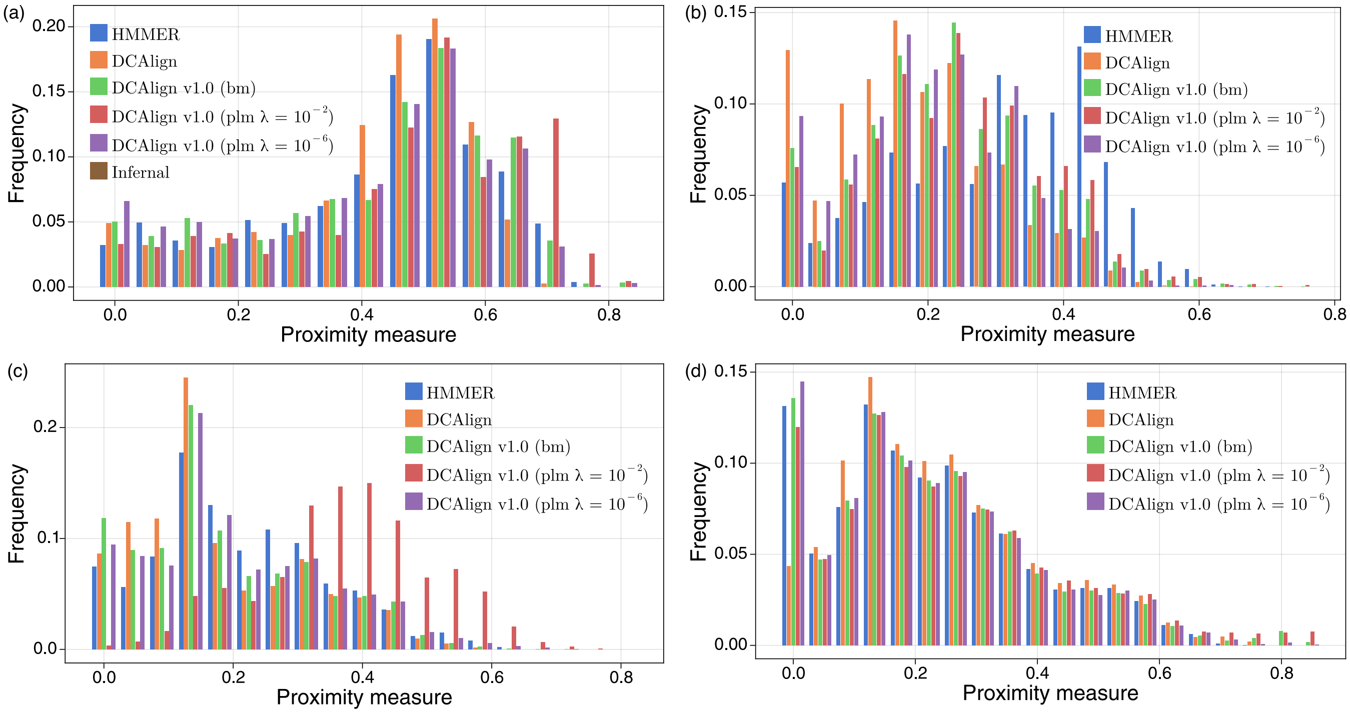

FIG. S5. Histogram of proximity measures as defined in Eq. 17 for the protein families PF00035, PF00677, PF00684, and PF00763 in panels (a), (b), (c), and (d) respectively. Each line corresponds to a different multiple sequence alignment obtained by `HMMER`, `DCAlign`, `DCAlign v1.0`. For the latter only, we provide several curves depending on the seed model used to align the query sequences. We use (bm) or (plm) when the model is trained by Boltzmann machine learning or pseudo-likelihood maximization respectively. For (plm) we vary the parameter λ associated with the regularization of the couplings and fields of the Potts model. All empirical distributions quite overlap for all the considered families, except the proximity measures of `DCAlign v1.0` (plm $\lambda = 10^{-2}$) for PF00684 and `HMMER` ones for PF00677. It is worth noting that DCA-based methods are more likely to show the highest peak in the first bin (corresponding to distances close to 0) suggesting that there exist more sequences in these alignments that are close to the seed than in any other alignment. The introduction of the prior over the Δn only slightly modifies the behavior of the first implementation, in some cases decreasing, in others increasing the average distance (see Tab. S9 for the mean and the standard deviation of the distribution).

Identifier	Mean $d\left(\boldsymbol{S}^{\mathrm{MSA}}, \boldsymbol{S}^{\mathrm{seed}}\right)$		Std $d\left(\boldsymbol{S}^{\mathrm{MSA}}, \boldsymbol{S}^{\mathrm{seed}}\right)$	
PF00035	HMMER	0.461	HMMER	0.190
	DCAlign	0.446	DCAlign	0.175
	DCAlign v1.0 (bm)	0.456	DCAlign v1.0 (bm)	0.200
	DCAlign v1.0 (plm)	0.505 ($\lambda = 10^{-2}$)	DCAlign v1.0 (plm)	0.202 ($\lambda = 10^{-2}$)
		**0.439** ($\lambda = 10^{-6}$)		0.205 ($\lambda = 10^{-6}$)
PF00677	HMMER	0.319	HMMER	0.149
	DCAlign	**0.195**	DCAlign	0.123
	DCAlign v1.0 (bm)	0.245	DCAlign v1.0 (bm)	0.130
	DCAlign v1.0 (plm)	0.260 ($\lambda = 10^{-2}$)	DCAlign v1.0 (plm)	0.133 ($\lambda = 10^{-2}$)
		0.220 ($\lambda = 10^{-6}$)		0.123 ($\lambda = 10^{-6}$)
PF00684	HMMER	0.237	HMMER	0.135
	DCAlign	**0.203**	DCAlign	0.130
	DCAlign v1.0 (bm)	0.209	DCAlign v1.0 (bm)	0.137
	DCAlign v1.0 (plm)	0.396 ($\lambda = 10^{-2}$)	DCAlign v1.0 (plm)	0.132 ($\lambda = 10^{-2}$)
		0.224 ($\lambda = 10^{-6}$)		0.141 ($\lambda = 10^{-6}$)
PF00763	HMMER	0.245	HMMER	0.166
	DCAlign	0.269	DCAlign	0.158
	DCAlign v1.0 (bm)	0.250	DCAlign v1.0 (bm)	0.178
	DCAlign v1.0 (plm)	0.268 ($\lambda = 10^{-2}$)	DCAlign v1.0 (plm)	0.190 ($\lambda = 10^{-2}$)
		**0.243** ($\lambda = 10^{-6}$)		0.172 ($\lambda = 10^{-6}$)

TABLE S9. Mean and standard deviation of the distance distribution between the multiple sequence alignments of PF00035, PF00677, PF00684, and PF00763 and the corresponding seed sequences as computed in Eq. 17. The associated histograms are reported in Fig. S5.

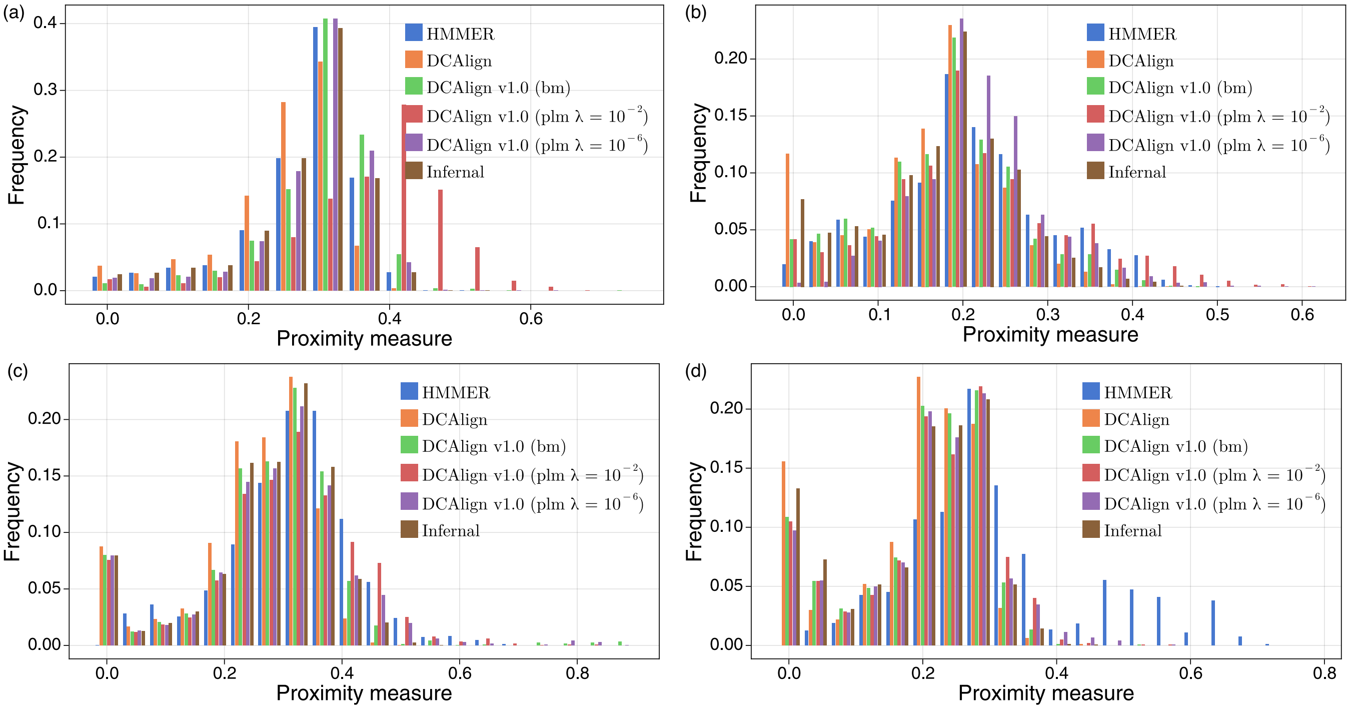

FIG. S6. Histogram of proximity measures as defined in Eq. 17 for the RNA families RF00059, RF00162, RF00167, and RF01734 in panels (a), (b), (c), and (d) respectively. Each line corresponds to a different multiple sequence alignment obtained by `HMMER`, `Infernal`, `DCAlign`, `DCAlign v1.0`. For the latter only, we provide several curves depending on the seed model used to align the query sequences. We use (bm) or (plm) when the model is trained by Boltzmann machine learning or pseudo-likelihood maximization respectively. For (plm) we vary the parameter λ associated with the regularization of the couplings and fields of the Potts model. All empirical distributions quite overlap for all the considered families, except the proximity measures of `DCAlign v1.0` (plm $\lambda = 10^{-2}$) for RF00059 (see panel (a)) and `HMMER` ones for RF01734 (see panel (d)). It is worth noting that DCA-based methods and `Infernal` are more likely to show the highest peak in the first bin (corresponding to distances close to 0), especially the first implementation `DCAlign`, suggesting that there exist more sequences in these alignments that are close to the seed than in any other alignment. The introduction of the prior over the Δn slightly modifies the behavior of the first implementation, mostly increasing the average distance (see Table S10 for the mean value and the standard deviation of the distribution).

Identifier	Mean $d\left(\boldsymbol{S}^{\mathrm{MSA}}, \boldsymbol{S}^{\mathrm{seed}}\right)$		Std $d\left(\boldsymbol{S}^{\mathrm{MSA}}, \boldsymbol{S}^{\mathrm{seed}}\right)$	
RF00059	HMMER	0.303	HMMER	0.086
	DCAlign	**0.275**	DCAlign	0.086
	DCAlign v1.0 (bm)	0.326	DCAlign v1.0 (bm)	0.077
	DCAlign v1.0 (plm)	0.398 ($\lambda = 10^{-2}$)	DCAlign v1.0 (plm)	0.108 ($\lambda = 10^{-2}$)
		0.316 ($\lambda = 10^{-6}$)		0.082 ($\lambda = 10^{-6}$)
	Infernal	0.302	Infernal	0.088
RF00162	HMMER	0.225	HMMER	0.097
	DCAlign	**0.173**	DCAlign	0.089
	DCAlign v1.0 (bm)	0.196	DCAlign v1.0 (bm)	0.089
	DCAlign v1.0 (plm)	0.228 ($\lambda = 10^{-2}$)	DCAlign v1.0 (plm)	0.110 ($\lambda = 10^{-2}$)
		0.233 ($\lambda = 10^{-6}$)		0.076 ($\lambda = 10^{-6}$)
	Infernal	0.187	Infernal	0.089
RF00167	HMMER	0.340	HMMER	0.108
	DCAlign	**0.270**	DCAlign	0.110
	DCAlign v1.0 (bm)	0.295	DCAlign v1.0 (bm)	0.128
	DCAlign v1.0 (plm)	0.316 ($\lambda = 10^{-2}$)	DCAlign v1.0 (plm)	0.135 ($\lambda = 10^{-2}$)
		0.305 ($\lambda = 10^{-6}$)		0.134 ($\lambda = 10^{-6}$)
	Infernal	0.290	Infernal	0.114
RF01734	HMMER	0.342	HMMER	0.142
	DCAlign	**0.201**	DCAlign	0.102
	DCAlign v1.0 (bm)	0.213	DCAlign v1.0 (bm)	0.099
	DCAlign v1.0 (plm)	0.223 ($\lambda = 10^{-2}$)	DCAlign v1.0 (plm)	0.105 ($\lambda = 10^{-2}$)
		0.224 ($\lambda = 10^{-6}$)		0.106 ($\lambda = 10^{-6}$)
	Infernal	0.204	Infernal	0.106

TABLE S10. Mean and standard deviation of the distance distribution between the multiple sequence alignments of RF00059, RF00162, RF00167, and RF01734 and the corresponding seed sequences as computed in Eq. 17. The associated histograms are reported in Fig. S6.

D. Leave-one-out simulations

1. BAliBASE

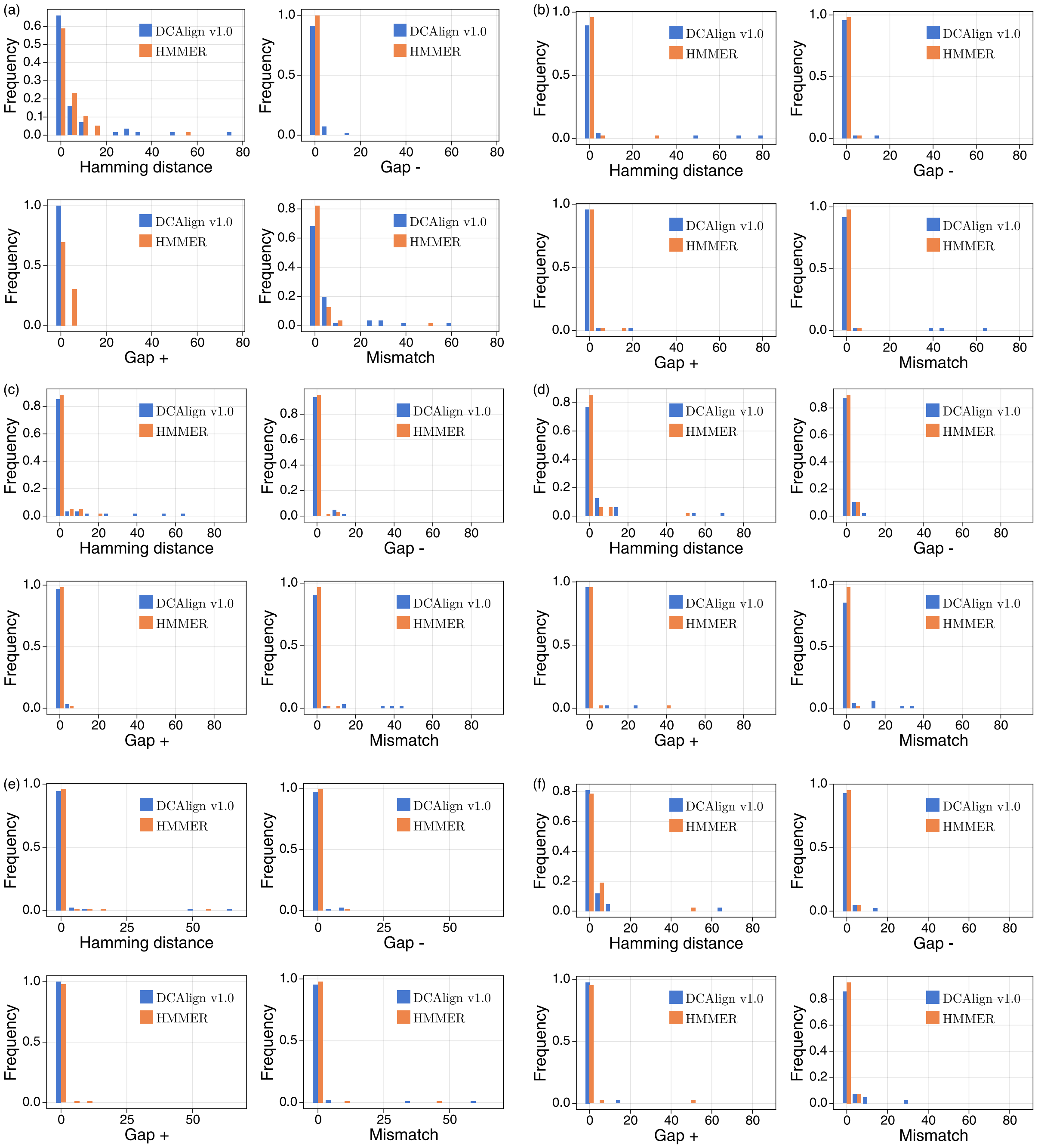

FIG. S7. Results for the leave-one-out experiment related to a subset of the families in the Reference set 2 of the BAliBASE dataset (see [16] for additional details). In these experiments, for a set of M sequences, we iteratively remove one sequence and we train the algorithms using the remaining $M-1$. We then align the excluded sequence and compute the Hamming distance between the ground truth and the aligned counterpart. To understand the nature of the divergence between the true and the aligned sequence, we also consider three auxiliary measures: *Gap - (+)* quantifies the number of gaps that were present (absent) in each column of the true sequence and that are replaced with matched symbols (gaps) in the aligned one. *Mismatch* counts the number of positions such that in both sequences no gap appears but they align to different residues. In these plots, we show the histograms of the four distances computed for the overall set of query sequences for different benchmark families: in (a), (b), (c), (d), (e), and (f) we show the results for BBS20008, BBS20030, BBS20032, BBS20033, BBS20036, and BBS20038 respectively. The more the mass of the empirical distributions is shifted to lower values of the metrics, the more the alignments are close to the ground truth.
The label `DCAlign v1.0` refers to the new implementation of our alignment method where the Potts Hamiltonian is learned through Pseudo-likelihood maximization setting $\lambda = 10^{-6}$. The comparison with Boltzmann machine-learned parameters is not contemplated because of the large computational time necessary to train a Boltzmann machine for each training set.
The mean values of each metric, and associated the standard errors, are reported in Table S11.

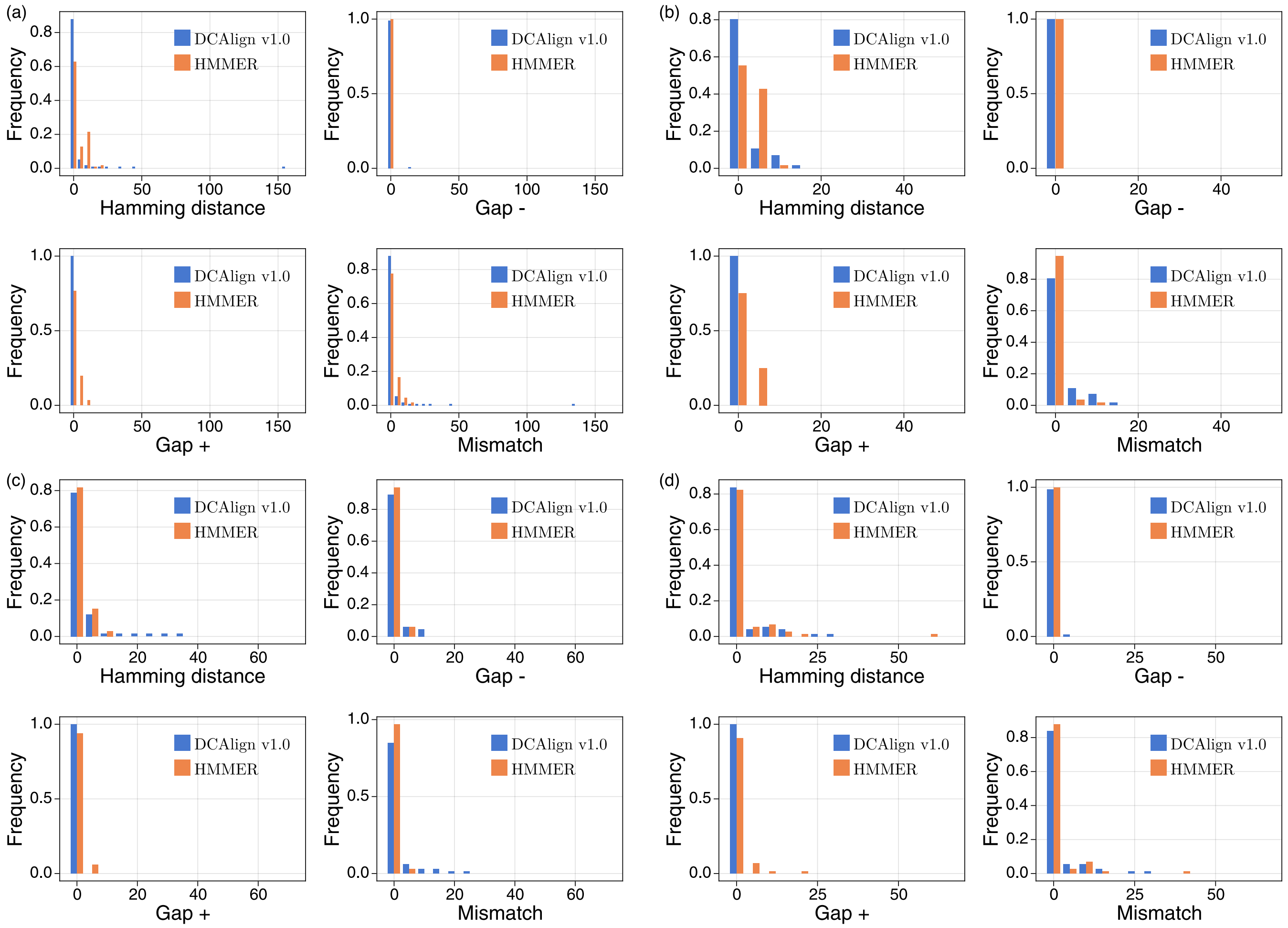

FIG. S8. Results for the leave-one-out experiment related to a subset of the families in the Reference set 3 of the BAliBASE dataset (see [16] for additional details). In these experiments, for a set of M sequences, we iteratively remove one sequence and we train the algorithms using the remaining $M-1$. We then align the excluded sequence and compute the Hamming distance between the ground truth and the aligned counterpart. To understand the nature of the divergence between the true and the aligned sequence, we also consider three auxiliary measures: *Gap - (+)* quantifies the number of gaps that were present (absent) in each column of the true sequence and that are replaced with matched symbols (gaps) in the aligned one. *Mismatch* counts the number of positions such that in both sequences no gap appears but they align to different residues. In these plots, we show the histograms of the four distances computed for the overall set of query sequences for different benchmark families: in (a), (b), (c), and (d) we show the results for BBS30001, BBS30020, BBS30022, and BBS30025 respectively. The more the mass of the empirical distributions is shifted to lower values of the metrics, the more the alignments are close to the ground truth.

The label `DCAlign v1.0` refers to the new implementation of our alignment method where the Potts Hamiltonian is learned through Pseudo-likelihood maximization setting $\lambda = 10^{-6}$. The comparison with Boltzmann machine-learned parameters is not contemplated because of the large computational time that one would necessitate to train a Boltzmann machine for each training set.

The mean values of each metric, and the associated standard errors, are reported in Table S11.

	BBS20008				
	f_{exact}	HD (mean, std err)	G+ (mean, std err)	G- (mean, std err)	M (mean, std err)
DCAlign v1.0	**0.554**	(6.857, 1.897)	(0.071, 0.035)	(0.946, 0.343)	(5.840, 1.620)
HMMER	0.071	(**5.875**, 1.105)	(3.089, 0.326)	(0.375, 0.107)	(2.411, 0.982)

	BBS20030				
	f_{exact}	HD (mean, std err)	G+ (mean, std err)	G- (mean, std err)	M (mean, std err)
DCAlign v1.0	**0.830**	(4.893, 2.520)	(0.723, 0.541)	(0.574, 0.399)	(3.596, 1.852)
HMMER	0.745	(**1.149**, 0.682)	(0.702, 0.363)	(0.255, 0.201)	(0.191, 0.141)

	BBS20032				
	f_{exact}	HD (mean, std err)	G+ (mean, std err)	G- (mean, std err)	M (mean, std err)
DCAlign v1.0	**0.590**	(4.574, 1.613)	(0.328, 0.174)	(1.344, 0.445)	(0.311, 1.215)
HMMER	0.098	(**4.344**, 1.583)	(3.066, 1.541)	(0.967, 0.306)	(0.311, 0.208)

	BBS20033				
	f_{exact}	HD (mean, std err)	G+ (mean, std err)	G- (mean, std err)	M (mean, std err)
DCAlign v1.0	**0.708**	(4.854, 1.931)	(0.792, 0.564)	(1.208, 0.423)	(2.854, 1.150)
HMMER	0.104	(**3.167**, 1.067)	(2.229, 0.883)	(0.750, 0.316)	(0.188, 0.132)

	BBS20036				
	f_{exact}	HD (mean, std err)	G+ (mean, std err)	G- (mean, std err)	M (mean, std err)
DCAlign v1.0	**0.802**	(1.791, 0.931)	(0.021, 0.015)	(0.429, 0.209)	(1.341, 0.776)
HMMER	0.571	(**1.615**, 0.681)	(0.769, 0.177)	(0.208, 0.136)	(0.637, 0.506)

	BBS20038				
	f_{exact}	HD (mean, std err)	G+ (mean, std err)	G- (mean, std err)	M (mean, std err)
DCAlign v1.0	**0.595**	(**3.429**, 1.601)	(0.405, 0.357)	(1.023, 0.496)	(2.000, 0.879)
HMMER	0.000	(3.905, 1.275)	(2.620, 1.162)	(0.548, 0.232)	(0.738, 0.303)

	BBS30001				
	f_{exact}	HD (mean, std err)	G+ (mean, std err)	G- (mean, std err)	M (mean, std err)
DCAlign v1.0	**0.819**	(**3.284**, 1.461)	(0.026, 0.015)	(0.301, 0.144)	(2.957, 1.323)
HMMER	0.000	(5.724, 0.459)	(3.621, 0.231)	(0.112, 0.034)	(1.991, 0.364)

	BBS30020				
	f_{exact}	HD (mean, std err)	G+ (mean, std err)	G- (mean, std err)	M (mean, std err)
DCAlign v1.0	**0.696**	(**2.036**, 0.536)	(0.000, 0.000)	(0.071, 0.071)	(1.964, 0.511)
HMMER	0.125	(3.839, 0.398)	(2.857, 0.273)	(0.000, 0.000)	(0.982, 0.288)

	BBS30022				
	f_{exact}	HD (mean, std err)	G+ (mean, std err)	G- (mean, std err)	M (mean, std err)
DCAlign v1.0	**0.667**	(3.288, 0.954)	(0.030, 0.021)	(1.030, 0.345)	(2.227, 0.686)
HMMER	0.106	(**2.424**, 0.309)	(1.591, 0.178)	(0.606, 0.222)	(0.227, 0.155)

	BBS30025				
	f_{exact}	HD (mean, std err)	G+ (mean, std err)	G- (mean, std err)	M (mean, std err)
DCAlign v1.0	**0.743**	(**2.446**, 0.693)	(0.000, 0.000)	(0.162, 0.092)	(2.288, 0.666)
HMMER	0.000	(4.554, 0.941)	(2.770, 0.308)	(0.122, 0.054)	(1.662, 0.668)

TABLE S11. Summary statistics of the histograms plotted in Figs. S7 and S8. In particular, for each of the families under consideration, we show the mean value and the standard error associated with the four metrics used as a comparison, i.e. the *Hamming distance* (HD), the *Gap +* (G+), the *Gap -* (G-), and the *Mismatches* (M). Additionally, we report the fraction of sequences f_{exact} that were perfectly aligned by the two methods used in this experiment (`HMMER` and `DCAlign v1.0`). Bold numbers are related to the best performances in terms of the Hamming distance (HD) and the f_{exact}. Although `DCAlign v1.0` always shows a larger f_{exact} than `HMMER`, suggesting that it likely aligns the test sequence with no error, `HMMER` attains the smallest mean value of the Hamming distance. This indicates that `DCAlign v1.0` generally perfectly aligns the sequences when it founds the correct envelope but it makes more mistakes than `HMMER` in the opposite situations, as underlined by the large value of the mean Mismatches (even though this is signaled by a large value of the Potts energy of the solution. See Fig. S9). On the contrary, `HMMER` often returns sequences with a few errors, which often consist of gaps (see the average value of the G+ measure).

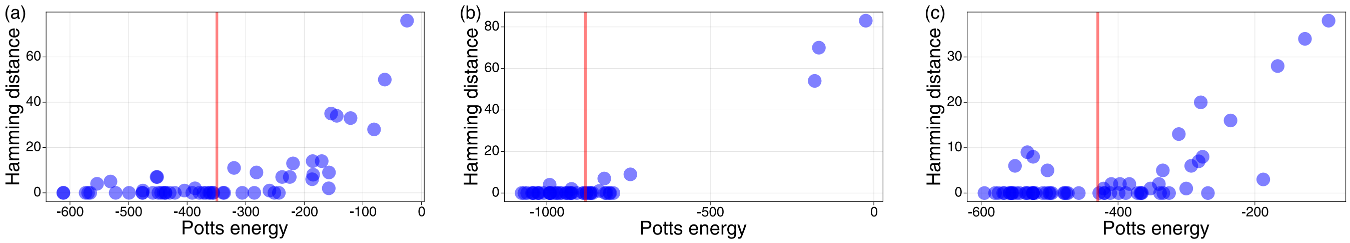

FIG. S9. Scatter plot of the Hamming distances between each ground truth and DCA-aligned sequence against the energy of the aligned sequences according to the Potts model used by `DCAlign v1.0`. The panels (a), (b), and (c) report the results for the datasets BBS20008, BBS20030, and BBS30022 respectively. Note that the value of the Potts energy positively correlates with the Hamming distance suggesting that the misaligned sequences are signaled with large energy, i.e. a low alignment score, often significantly larger than its expected value (shown as a red vertical line in all panels).

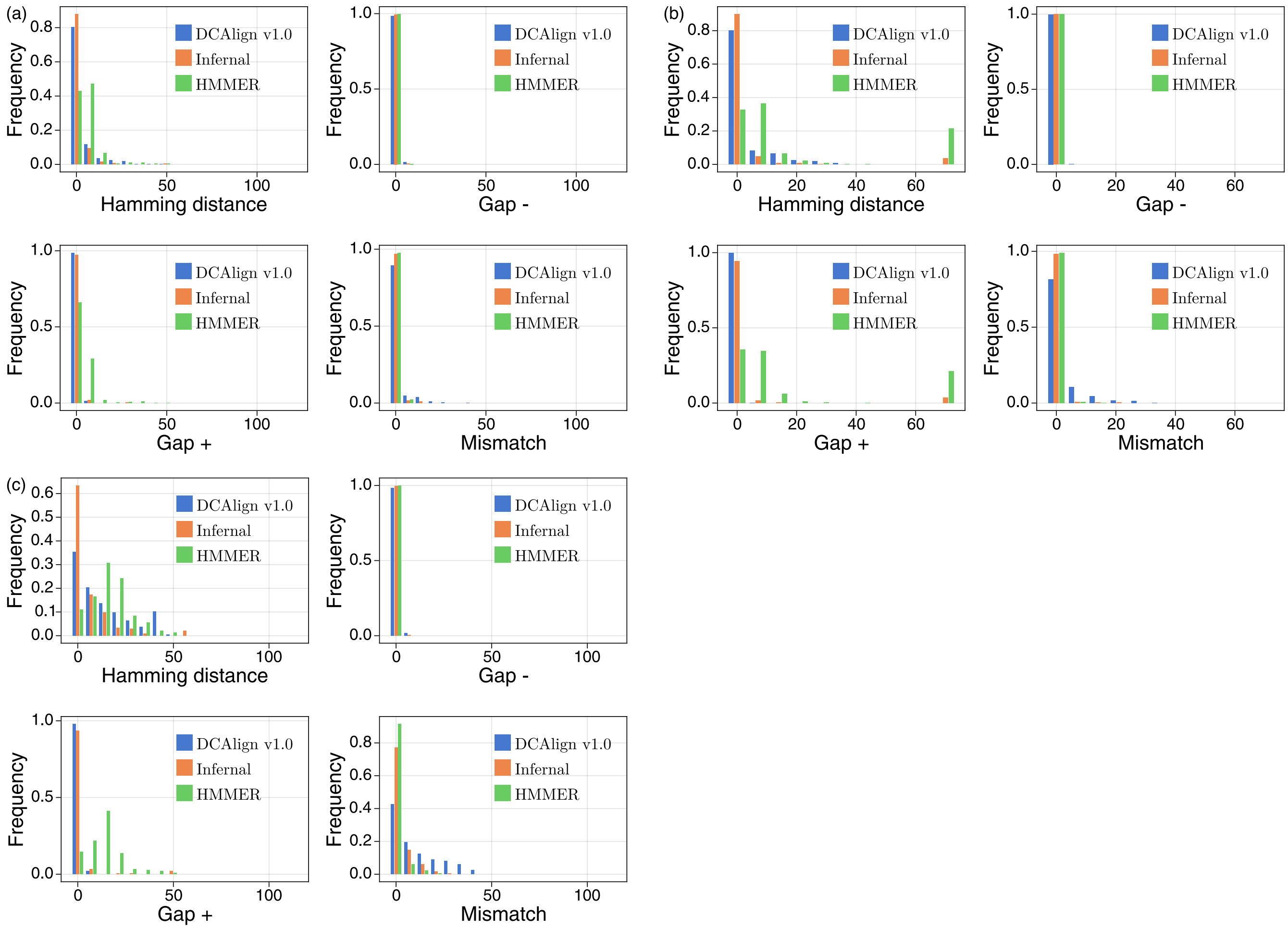

FIG. S10. Results of the leave-one-out experiment for the RNA families *tRNA*, *rRNA*, and *U5* of the BRaliBase III dataset [7]. In these experiments, for a set of M sequences, we iteratively remove one sequence and we train the algorithms using the remaining $M-1$. We then align the excluded sequence using `HMMER`, `Infernal`, or `DCAlign v1.0` and compute the Hamming distance between the ground truth and the aligned counterpart. To understand the nature of the divergence between the true and the aligned sequence, we also consider three auxiliary measures: *Gap - (+)* quantifies the number of gaps that were present (absent) in each column of the true sequence and that are replaced with matched symbols (gaps) in the aligned one. *Mismatch* counts the number of positions such that in both sequences no gap appears but they align to different residues. Panels (a), (b), and (c) show the results for rRNA, tRNA, and U5 respectively. The more the mass of the empirical distributions is shifted to lower values of the metrics, the more the alignments are close to the ground truth.
The label `DCAlign v1.0` refers to the new implementation of our alignment method where the Potts Hamiltonian is learned through Pseudo-likelihood maximization setting $\lambda = 10^{-2}$. The mean values of each metric, and the associated standard errors, are reported in Table S12.
When `Infernal` or `HMMER` do not recognize the domain - this happens when no sequence has an e-value larger than the default threshold - we consider as an aligned sequence a series of gaps to easily determine how frequently this occurs. In panel (b) we see a peak in the Hamming distance (for `HMMER` and `Infernal`) in correspondence with the length $L = 72$; this peak recurs in the Gap + metric suggesting that for these sequences `HMMER` and `Infernal` did not find a reasonable domain to align.
To train Infernal, we use the knowledge of the secondary structure differently from `HMMER` and `DCAlign v1.0` which exploit only the information provided by the seed alignment.

	rRNA				
	f_{exact}	HD (mean, std err)	G+ (mean, std err)	G- (mean, std err)	M (mean, std err)
DCAlign v1.0	0.512	(3.748, 0.278)	(0.638, 0.059)	(0.912, 0.066)	(2.198, 0.207)
HMMER	0.000	(8.427, 0.264)	(6.651, 0.214)	(0.902, 0.056)	(0.874, 0.073)
Infernal	**0.538**	(**2.453**, 0.198)	(0.902, 0.097)	(0.797, 0.054)	(0.754, 0.093)
	tRNA				
	f_{exact}	HD (mean, std err)	G+ (mean, std err)	G- (mean, std err)	M (mean, std err)
DCAlign v1.0	0.629	(**3.799**, 0.225)	(0.274, 0.023)	(0.561, 0.031)	(2.964, 0.195)
HMMER	0.000	(21.976, 0.794)	(21.454, 0.800)	(0.252, 0.019)	(0.269, 0.038)
Infernal	**0.709**	(3.926, 0.411)	(3.211, 0.406)	(0.241, 0.018)	(0.475, 0.063)
	U5				
	f_{exact}	HD (mean, std err)	G+ (mean, std err)	G- (mean, std err)	M (mean, std err)
DCAlign v1.0	0.128	(15.630, 0.913)	(1.281, 0.118)	(1.566, 0.119)	(12.783, 0.827)
HMMER	0.000	(19.332, 0.663)	(16.217, 0.586)	(0.719, 0.061)	(2.396, 0.236)
Infernal	**0.281**	(**7.583**, 0.701)	(2.719, 0.541)	(1.081, 0.086)	(3.783, 0.385)

TABLE S12. Summary statistics associated with the histograms in Fig. S10. In the columns of this table, we report the mean value and the standard error of the four considered metrics, i.e. the Hamming distance, Gap +, Gap - and Mismatches (see the caption of Fig. S10 for a definition of these quantities), for the four families, rRNA, tRNA, and U5. The lower the values, the better the alignment. We also report the value of f_{exact} which is the fraction of sequences that are perfectly detected by the three considered methods (`HMMER`, `Infernal`, and `DCAlign v1.0`). Larger values of f_{exact} correspond to a larger number of perfect alignments. Bold numbers are associated with the best performance achieved in terms of f_{exact} and the Hamming distance. Although `Infernal` mostly outperforms all other methods, it is worth noting that `DCAlign v1.0` achieves very similar values of the two reference metrics.

3. PH domain

	PF00062				
	f_{exact}	HD (mean, std err)	G+ (mean, std err)	G- (mean, std err)	M (mean, std err)
DCAlign v1.0	**0.234**	(**48.285**, 1.140)	(1.675, 0.097)	(1.210, 0.115)	(45.400, 1.107)
HMMER	0.167	(58.112, 1.075)	(5.656, 0.600)	(1.070, 0.152)	(51.386, 1.036)
	PF00182				
	f_{exact}	HD (mean, std err)	G+ (mean, std err)	G- (mean, std err)	M (mean, std err)
DCAlign v1.0	**0.333**	(8.185, 5.934)	(1.111, 0.195)	(1.407, 1.075)	(5.667, 4.834)
HMMER	0.037	(**6.296**, 2.843)	(3.370, 2.143)	(0.444, 0.209)	(2.481, 0.703)
	PF00638				
	f_{exact}	HD (mean, std err)	G+ (mean, std err)	G- (mean, std err)	M (mean, std err)
DCAlign v1.0	**0.469**	(**82.898**, 11.698)	(0.122, 0.086)	(0.041, 0.029)	(82.735, 11.673)
HMMER	0.000	(105.061, 6.704)	(5.898, 3.737)	(0.061, 0.035)	(99.102, 6.877)
	PF00640				
	f_{exact}	HD (mean, std err)	G+ (mean, std err)	G- (mean, std err)	M (mean, std err)
DCAlign v1.0	**0.077**	(**14.051**, 3.003)	(3.897, 0.365)	(3.026, 1.257)	(7.128, 2.357)
HMMER	0.026	(18.564, 3.593)	(8.359, 2.315)	(2.051, 1.077)	(8.124, 2.182)
	PF01464				
	f_{exact}	HD (mean, std err)	G+ (mean, std err)	G- (mean, std err)	M (mean, std err)
DCAlign v1.0	**0.295**	(**24.967**, 3.823)	(0.328, 0.083)	(8.640, 1.532)	(17.607, 2.917)
HMMER	0.000	(35.033, 3.937)	(15.393, 3.343)	(2.033, 0.745)	(17.607, 2.979)
	PF09380				
	f_{exact}	HD (mean, std err)	G+ (mean, std err)	G- (mean, std err)	M (mean, std err)
DCAlign v1.0	**0.500**	(**14.300**, 4.422)	(0.150, 0.105)	(0.775, 0.725)	(13.375, 3.743)
HMMER	0.000	(17.700, 3.955)	(6.000, 2.704)	(0.175, 0.129)	(11.525, 1.656)

TABLE S13. Mean value and standard errors of the Hamming distance, Gap +, Gap , and Mismatch computed from the histogram in Fig. S11. The first columns of the table also show f_{exact}, the fraction of sequences perfectly reconstructed by the alignment methods under consideration (in this case, `DCAlign v1.0` and `HMMER`). In general, these alignments are probably more challenging than the datasets considered in the previous leave-one-out experiment. This emerges *a posteriori* by looking at the empirical distributions in Fig. S11 of the four metrics (and their statistics), which are way broader than those of the other datasets, and the value of the f_{exact} (Hamming distance) which is significantly smaller (larger, and comparable to L).

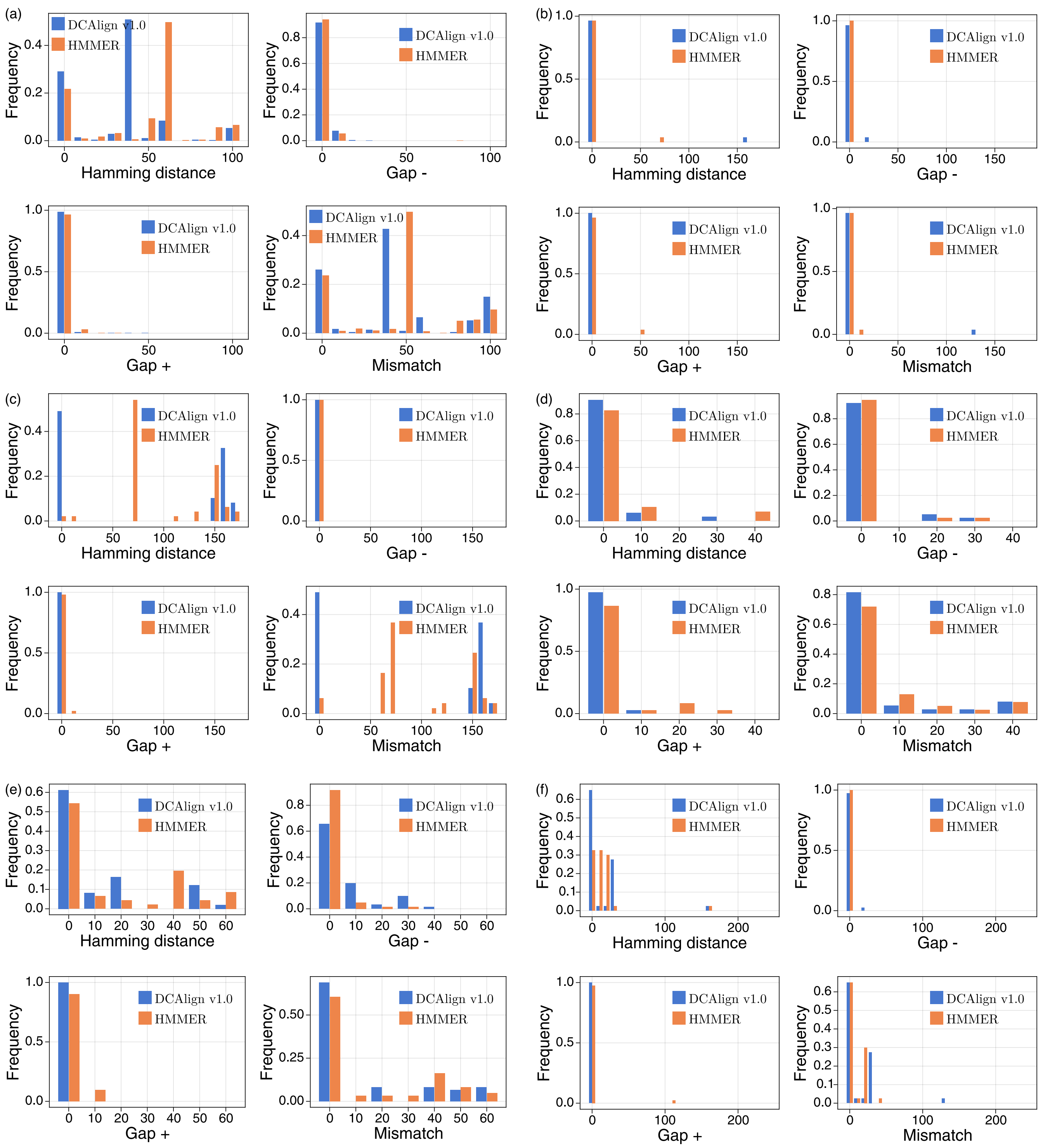

FIG. S11. In this plot we show the empirical distribution of the Hamming distance, Gap +, Gap -, and Mismatch for the leave-one-out test where the ground truths are determined by a set of multiple structural alignments. More precisely, we select from the clan CL0266 of Pfam, the most representative families - in terms of the number of sequences - having at least twenty known crystallized structures, available in the Protein Data Bank. These families are labeled as PF00062, PF00182, PF00638, PF00640, PF01464, and PF09380, and all contain PH domains. We aligned these structures using Caretta [1] which also outputs the multiple sequence alignment associated with them. These MSAs have a length L of hundreds of sites, most of them filled by insertions, unlikely the common MSAs used as seed alignments. For this reason, we annotate the columns by filtering those displaying a large fraction of insertions, identifying the gaps "-" and the insertions (lowercase symbols). This last step determines a final MSA (the final length is reported in Table S2) considered in this test as the ground truth.
As displayed in the histograms and suggested by the Table in S13,`DCAlign v1.0` outperforms `HMMER` showing for most of the families a smaller mean Hamming distance. The nature of the errors, quantified by Gap +, Gap -, and Mismatch, is slightly different with respect to the other datasets: while for `DCAlign v1.0` the contribution of the Mismatches is dominant as for the other cases, `HMMER` shows large values for both Gap + and Mismatch.

4. Other structural-based alignments

	PF00013				
	f_{exact}	HD (mean, std err)	G+ (mean, std err)	G- (mean, std err)	M (mean, std err)
DCAlign v1.0	**0.402**	(**19.326**, 2.366)	(0.772, 0.294)	(3.565, 0.844)	(14.990, 2.004)
HMMER	0.022	(47.739, 2.412)	(43.424, 2.673)	(0.130, 0.073)	(4.185, 1.140)
	PF09408				
	f_{exact}	HD (mean, std err)	G+ (mean, std err)	G- (mean, std err)	M (mean, std err)
DCAlign v1.0	**0.239**	(**21.487**, 0.483)	(0.135, 0.016)	(1.810, 0.117)	(19.542, 0.439)
HMMER	0.000	(39.552, 0.701)	(27.670, 0.842)	(0.407, 0.023)	(11.476, 0.312)
	IPR000008				
	f_{exact}	HD (mean, std err)	G+ (mean, std err)	G- (mean, std err)	M (mean, std err)
DCAlign v1.0	**0.152**	(**29.824**, 1.680)	(10.686, 0.746)	(2.828, 0.329)	(16.309, 0.984)
HMMER	0.000	(65.480, 1.638)	(61.142, 2.027)	(0.603, 0.139)	(3.735, 0.564)
	IPR000020				
	f_{exact}	HD (mean, std err)	G+ (mean, std err)	G- (mean, std err)	M (mean, std err)
DCAlign v1.0	**0.409**	(**18.295**, 3.048)	(0.068, 0.050)	(1.114, 0.455)	(17.114, 2.874)
HMMER	0.000	(35.955, 3.024)	(22.045, 3.180)	(0.477, 0.257)	(13.432, 2.618)
	IPR001466				
	f_{exact}	HD (mean, std err)	G+ (mean, std err)	G- (mean, std err)	M (mean, std err)
DCAlign v1.0	**0.346**	(**15.673**, 1.702)	(0.093, 0.017)	(0.953, 0.120)	(14.626, 1.618)
HMMER	0.000	(23.947, 1.451)	(6.872, 0.467)	(0.813, 0.088)	(16.262, 1.063)
	IPR001789				
	f_{exact}	HD (mean, std err)	G+ (mean, std err)	G- (mean, std err)	M (mean, std err)
DCAlign v1.0	**0.236**	(**23.170**, 1.110)	(0.211, 0.045)	(1.729, 0.207)	(21.229, 1.054)
HMMER	0.000	(54.048, 0.852)	(51.817, 1.013)	(0.092, 0.022)	(2.140, 0.274)

TABLE S14. Mean value and standard errors of the Hamming distance, Gap +, Gap , and Mismatch computed from the histogram in Fig. S12. The first columns of the table also show f_{exact}, the fraction of sequences perfectly reconstructed by the alignment methods under consideration (in this case, `DCAlign v1.0` and `HMMER`). Bold numbers are associated with the best performance for the corresponding task.

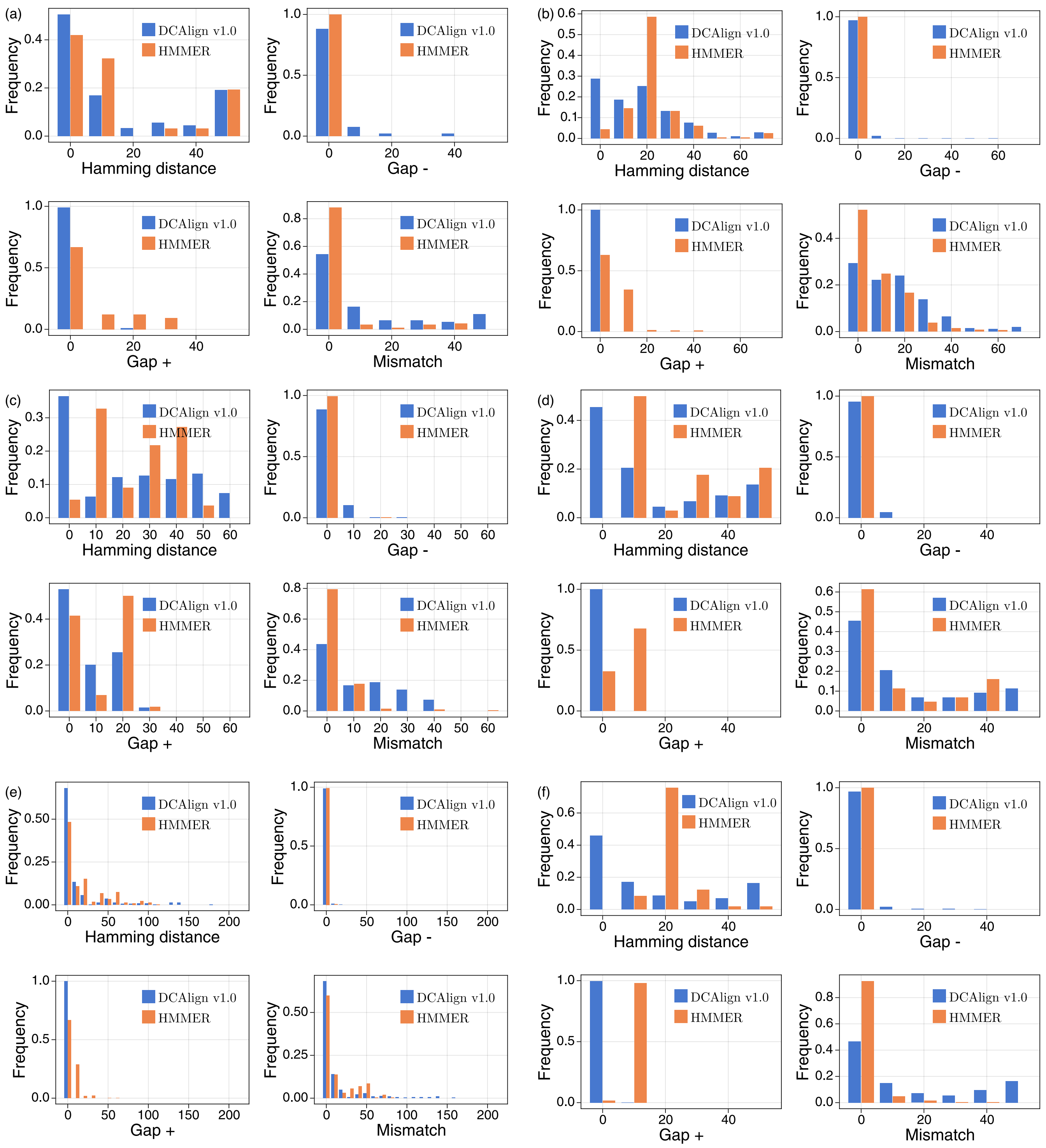

FIG. S12. In this plot we show the empirical distribution of the Hamming distance, Gap +, Gap -, and Mismatch for the leave-one-out test where the ground truths are determined by a set of multiple structural alignments. We select from InterPro, six protein domains having at least a reasonable number of known crystallized structures (on average a few hundred), available in the Protein Data Bank. These families are labeled as PF00013, PF09408, IPR000008, IPR000020, IPR001466, and IPR001789 and correspond to the KH domain, Betacoronavirus spike glycoprotein S1, C2 domain, Anaphylatoxin/fibulin, Beta-lactamase-related, and the response regulator, receiver domain respectively. We aligned these structures using Caretta [1] which also outputs the multiple sequence alignment associated with them. These MSAs have a length L of hundreds of sites, most of them filled by insertions, unlikely the common MSAs used as seed alignments. For this reason, we annotate the columns by filtering those displaying a large fraction of insertions, identifying the gaps "-" and the insertions (lowercase symbols). This last step determines a final MSA (the final length is reported in Table S2) considered in this test as the ground truth.
As displayed in the histograms and suggested by the Table in S14, `DCAlign v1.0` outperforms `HMMER` showing for most of the families a smaller mean Hamming distance. The nature of the errors, quantified by Gap +, Gap -, and Mismatch, is slightly different with respect to the other datasets: while for `DCAlign v1.0` the contribution of the Mismatches is dominant as for the other cases, `HMMER` shows large values for both Gap + and Mismatch.

E. Detection of distant sequences

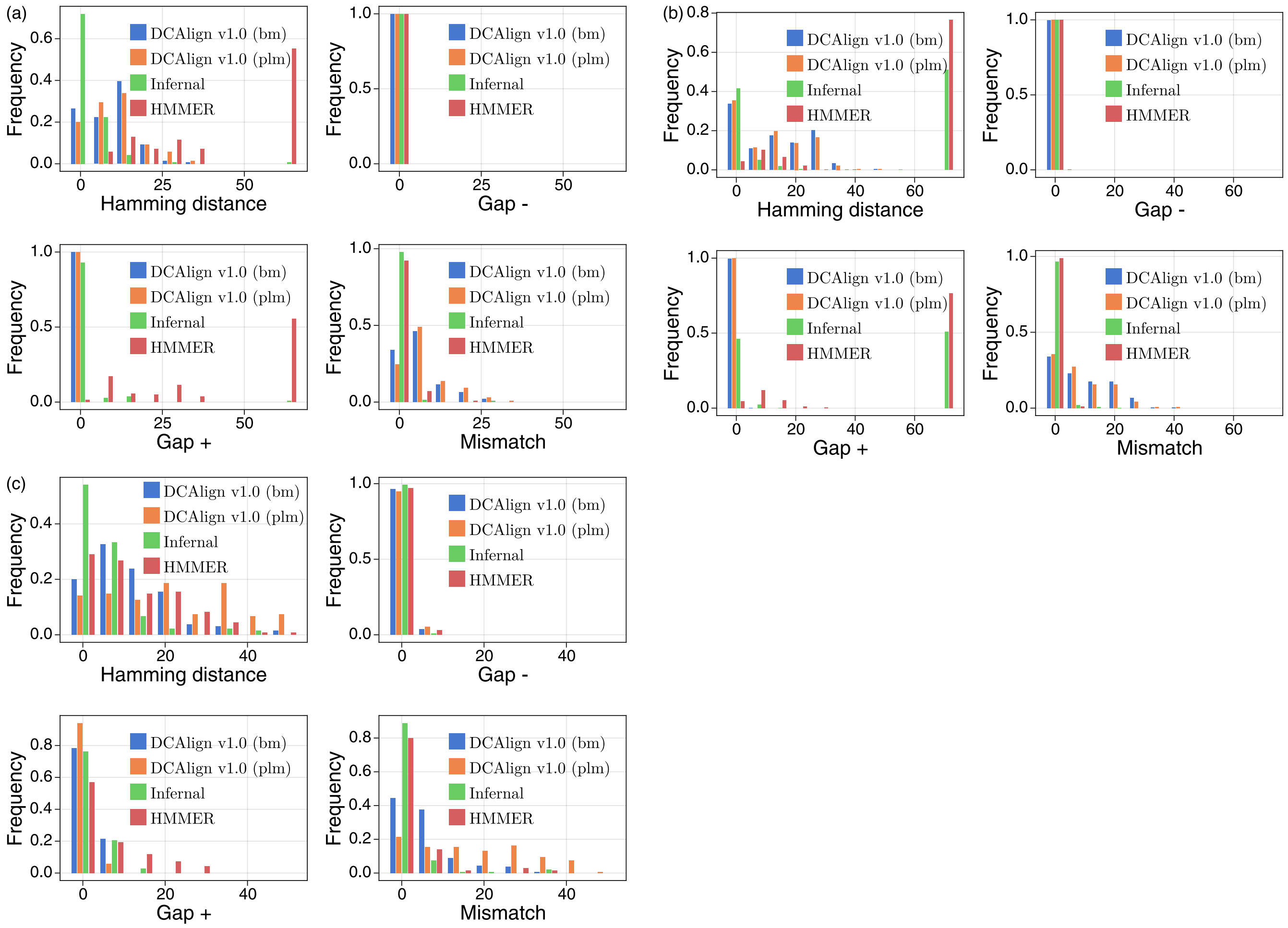

FIG. S13. For this experiment we split into a training and test set the datasets selected in [17]. i.e. type P1, tRNA, and the seed of the RNA family RF00162 (see Table S6). We first perform a single-linkage clustering of the sequences and we create two sets such that the Hamming distance between each possible couple of sequences (one taken from the first set, and one from the second one) is larger than 30 % of the length L (in one case 25%, see the Table in S15). We train `HMMER`, `Infernal`, `DCAlign v1.0` (using both pseudo-likelihood maximization or Boltzmann machine learning) using the training set and we align the so-defined test sequences. In this plot, we show the statistics of the four distance metrics: Hamming distance, Gap +, Gap -, and Mismatch; panels (a), (b), and (c) are associated with type P1, tRNA, and RF00162 seed. When `Infernal` or `HMMER` do not recognize the domain - this happens when no sequence has an e-value larger than the default threshold - we consider as an aligned sequence a series of gaps to easily determine how frequently this occurs. For instance, in panel (a) we see a peak in the Hamming distance (for `HMMER`) in correspondence with the length $L = 65$; this peak recurs in the Gap + metric suggesting that for these sequences `HMMER` did not find an appropriate domain to align. It is worth noting that the two outperforming methods, showing a large portion of the distribution concentrated at small distances, are `Infernal` and `DCAlign v1.0`; this is confirmed by the statistics shown in Table S15.

	typeP1 (sequence identity < 0.70)				
	f_{exact}	HD (mean, std err)	G+ (mean, std err)	G- (mean, std err)	M(mean, std err)
DCAlign v1.0 (bm)	0.029	(12.986, 0.630)	(1.453, 0.085)	(1.547, 0.117)	(9.986, 0.584)
DCAlign v1.0 (plm)	0.029	(14.194, 0.688)	(1.273, 0.087)	(1.511, 0.119)	(11.410, 0.644)
HMMER	0.000	(46.885, 1.791)	(44.871, 2.001)	(0.489, 0.078)	(1.523, 0.286)
Infernal	**0.173**	(**5.626**, 0.598)	(3.101, 0.524	(0.950, 0.126)	(1.576, 0.264)

	tRNA (sequence identity < 0.70)				
	f_{exact}	HD (mean, std err)	G+ (mean, std err)	G- (mean, std err)	M(mean, std err)
DCAlign v1.0 (bm)	0.263	(15.265, 0.589)	(1.575, 0.075)	(1.531, 0.074)	(12.159, 0.483)
DCAlign v1.0 (plm)	**0.274**	(**14.431**, 0.581)	(1.511, 0.071)	(1.531, 0.072)	(11.389, 0.474)
HMMER	0.000	(57.243, 1.181)	(56.982, 1.201)	(0.033, 0.011)	(0.228, 0.053)
Infernal	0.250	(37.681, 1.611)	(36.892, 1.644)	(0.212, 0.026)	(0577, 0.111)

	RF00162 seed (sequence identity < 0.75)				
	f_{exact}	HD (mean, std err)	G+ (mean, std err)	G- (mean, std err)	M(mean, std err)
DCAlign v1.0 (bm)	0.037	(14.681, 0.810)	(4.511, 0.223)	(1.289, 0.167)	(8.881, 0.677)
DCAlign v1.0 (plm)	0.037	(24.526, 1.291)	(2.748, 0.168)	(1.689, 0.191)	(20.089, 1.192)
HMMER	0.000	(15.037, 0.928)	(9.296, 0.676)	(1.356, 0.166)	(4.385, 0.606)
Infernal	**0.156**	(**7.615**, 0.730)	(4.081, 0.375)	(1.089, 0.140)	(2.444, 0.570)

TABLE S15. For each dataset, typeP1, tRNA, and the RF00162 seed, we report the fraction of perfectly aligned sequences f_{exact}, and the means and standard errors of the four considered distance-metrics: Hamming distance (HD), Gap + (G+), Gap - (G-), and Mismatch (M). Bold numbers refer to the best-performing methods in terms of f_{exact}, and Hamming distance. For two families out of three `Infernal` achieves the best values for the two main metrics, while `DCAlign v1.0 (plm)` shows the best alignment for the tRNA family. Note that `DCAlign v1.0 (bm)` performs similarly to the two methods. Surprisingly, the DCA-based methods can cope with the alignment of RNA domains, providing similar results to those given by `Infernal`, with no information about the secondary structure, which is instead used in the training of the covariance model linked to Infernal.

F. Running time

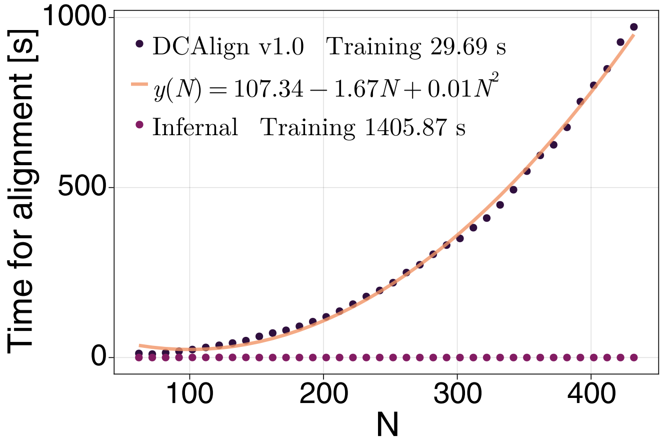

FIG. S14. Running time required by `DCAlign v.10` (dark purple dots) and `Infernal` (light purple dots) to align one of the sequences of the fluoride riboswitch family (RF01734), identified as `LZRT01000091.1`. The length of the unaligned sequence N varies in the range $[L+2, L+400]$. The computing time of `Infernal` is of the order of tenths of seconds independently of N, while the running time of `DCAlign v1.0` scales quadratically with N (see the fit curve $y(N)$). On the contrary, the training of the DCA-based model of the seed required only about 30 s against the 1406 s of the covariance model. In particular, the subroutine `cmcalibrate()` is significantly time-demanding. For the sake of fairness, we use a single thread while training and aligning through Infernal because `DCAlign v1.0` does not allow for multi-thread computations.

[1] Akdel, M., Durairaj, J., de Ridder, D., and van Dijk, A. D. (2020). Caretta - a multiple protein structure alignment and feature extraction suite. *Computational and Structural Biotechnology Journal*, **18**, 981–992.
[2] Bahr, A., Thompson, J. D., Thierry, J.-C., and Poch, O. (2001). BAliBASE (Benchmark Alignment dataBASE): enhancements for repeats, transmembrane sequences and circular permutations. *Nucleic Acids Research*, **29**(1), 323–326.
[3] Berman, H. M., Westbrook, J., Feng, Z., Gilliland, G., Bhat, T. N., Weissig, H., Shindyalov, I. N., and Bourne, P. E. (2000). The Protein Data Bank. *Nucleic Acids Research*, **28**(1), 235–242.
[4] Ekeberg, M., Lövkvist, C., Lan, Y., Weigt, M., and Aurell, E. (2013). Improved contact prediction in proteins: Using pseudolikelihoods to infer Potts models. *Physical Review E*, **87**(1), 012707. Publisher: American Physical Society.
[5] El-Gebali, S., Mistry, J., Bateman, A., Eddy, S. R., Luciani, A., Potter, S. C., Qureshi, M., Richardson, L. J., Salazar, G. A., Smart, A., Sonnhammer, E. L., Hirsh, L., Paladin, L., Piovesan, D., Tosatto, S. C., and Finn, R. D. (2019). The pfam protein families database in 2019. **47**, D427–D432.
[6] Feinauer, C. (2015). pdbtool: An object-oriented julia tool to parse pdb files and work with them.
[7] Freyhult, E. K., Bollback, J. P., and Gardner, P. P. (2007). Exploring genomic dark matter: A critical assessment of the performance of homology search methods on noncoding RNA. *Genome Research*, **17**(1), 117–125.
[8] Kalvari, I., Nawrocki, E. P., Ontiveros-Palacios, N., Argasinska, J., Lamkiewicz, K., Marz, M., Griffiths-Jones, S., Toffano-Nioche, C., Gautheret, D., Weinberg, Z., Rivas, E., Eddy, S. R., Finn, R., Bateman, A., and Petrov, A. I. (2020). Rfam 14: expanded coverage of metagenomic, viral and microRNA families. *Nucleic Acids Research*, **49**(D1), D192–D200.
[9] Mistry, J., Chuguransky, S., Williams, L., Qureshi, M., Salazar, G., Sonnhammer, E. L. L., Tosatto, S. C. E., Paladin, L., Raj, S., Richardson, L. J., Finn, R. D., and Bateman, A. (2021). Pfam: The protein families database in 2021. **49**, D412–D419.
[10] Muntoni, A. P., Pagnani, A., Weigt, M., and Zamponi, F. (2020). Aligning biological sequences by exploiting residue conservation and coevolution. *Physical Review E*, **102**(6), 062409. Publisher: American Physical Society.
[11] Muntoni, A. P., Pagnani, A., Weigt, M., and Zamponi, F. (2021). adabmDCA: adaptive Boltzmann machine learning for biological sequences. *BMC Bioinformatics*, **22**(1), 528.
[12] Paysan-Lafosse, T., Blum, M., Chuguransky, S., Grego, T., Pinto, B. L., Salazar, G., Bileschi, M., Bork, P., Bridge, A., Colwell, L., Gough, J., Haft, D., Letunić, I., Marchler-Bauer, A., Mi, H., Natale, D., Orengo, C., Pandurangan, A., Rivoire, C., Sigrist, C. J. A., Sillitoe, I., Thanki, N., Thomas, P. D., Tosatto, S. C. E., Wu, C., and Bateman, A. (2023). InterPro in 2022. **51**, D418–D427.
[13] Roth, A., Weinberg, Z., Chen, A. G. Y., Kim, P. B., Ames, T. D., and Breaker, R. R. (2014). A widespread self-cleaving ribozyme class is revealed by bioinformatics. *Nature Chemical Biology*, **10**(1), 56–60. Number: 1 Publisher: Nature Publishing Group.
[14] Sarti, E. and Pagnani, A. (2020). infernet-h2020/pfam_interactions: Initial release.
[15] Sprinzl, M., Horn, C., Brown, M., Ioudovitch, A., and Steinberg, S. (1998). Compilation of tRNA sequences and sequences of tRNA genes. *Nucleic Acids Research*, **26**(1), 148–153.
[16] Thompson, J. D., Koehl, P., Ripp, R., and Poch, O. (2005). BAliBASE 3.0: Latest developments of the multiple sequence alignment benchmark. *Proteins: Structure, Function, and Bioinformatics*, **61**(1), 127–136.
[17] Wilburn, G. W. and Eddy, S. R. (2020). Remote homology search with hidden Potts models. *PLOS Computational Biology*, **16**(11), e1008085. Publisher: Public Library of Science.
